# The Smc5/Smc6/MAGE Complex Confers Resistance to Caffeine and Genotoxic Stress in *Drosophila melanogaster*

**DOI:** 10.1371/journal.pone.0059866

**Published:** 2013-03-28

**Authors:** Xiao Li, Ran Zhuo, Stanley Tiong, Francesca Di Cara, Kirst King-Jones, Sarah C. Hughes, Shelagh D. Campbell, Rachel Wevrick

**Affiliations:** 1 Department of Medical Genetics, University of Alberta, Edmonton, Alberta, Canada; 2 Department of Biological Sciences, University of Alberta, Edmonton, Alberta, Canada; St. Georges University of London, United Kingdom

## Abstract

The SMC5/6 protein complex consists of the Smc5, Smc6 and Non-Smc-Element (Nse) proteins and is important for genome stability in many species. To identify novel components in the DNA repair pathway, we carried out a genetic screen to identify mutations that confer reduced resistance to the genotoxic effects of caffeine, which inhibits the ATM and ATR DNA damage response proteins. This approach identified inactivating mutations in *CG5524* and *MAGE*, homologs of genes encoding Smc6 and Nse3 in yeasts. The fact that *Smc5* mutants are also caffeine-sensitive and that Mage physically interacts with *Drosophila* homologs of Nse proteins suggests that the structure of the Smc5/6 complex is conserved in *Drosophila*. Although Smc5/6 proteins are required for viability in *S. cerevisiae*, they are not essential under normal circumstances in *Drosophila.* However, flies carrying mutations in *Smc5*, *Smc6* and *MAGE* are hypersensitive to genotoxic agents such as ionizing radiation, camptothecin, hydroxyurea and MMS, consistent with the Smc5/6 complex serving a conserved role in genome stability. We also show that mutant flies are not compromised for pre-mitotic cell cycle checkpoint responses. Rather, caffeine-induced apoptosis in these mutants is exacerbated by inhibition of ATM or ATR checkpoint kinases but suppressed by Rad51 depletion, suggesting a functional interaction involving homologous DNA repair pathways that deserves further scrutiny. Our insights into the SMC5/6 complex provide new challenges for understanding the role of this enigmatic chromatin factor in multi-cellular organisms.

## Introduction

The evolutionarily conserved Structural Maintenance of Chromosomes proteins are essential for the organization, segregation, and stability of the genome [1], [2], [3]. Three functionally distinct SMC complexes have been defined in eukaryotes: cohesin (Smc1/3), condensin (Smc2/4), and the otherwise unnamed Smc5/6 complex, each accompanied by a unique set of regulatory subunits. Cohesin holds sister chromatids together after DNA replication and plays important roles in regulation of gene expression and DNA repair [4], while condensin is essential for mitotic chromosome organization and segregation [5]. The Smc5/6 complex is less well characterized but is required for homologous DNA recombination-based processes, including repair of DNA double strand breaks, restart of stalled replication forks, ribosomal DNA maintenance, telomere elongation, and chromosome dynamics during meiosis [6], [7], [8], [9], [10].

The Smc5/6 complex in the yeasts is made up of eight subunits that form three sub-complexes: Smc6-Smc5-Nse2, Nse1-Nse3-Nse4, and Nse5-Nse6 [11]. Smc5 and Smc6 dimerize through their hinge regions to form the core. The Sumo ligase Nse2 associates with the Smc5-Smc6 heterodimer through a direct interaction with Smc5 [12], [13], [14]. Nse1, a RING finger protein with E3 ubiquitin ligase activity, Nse4, the kleisin component of the complex, and Nse3, a MAGE homolog, interact with each other to form the sub-complex that bridges the head domain of the Smc5-Smc6 heterodimer [7], [14], [15], [16], [17]. Nse5 and Nse6 form the third sub-complex in yeasts, but these proteins have no counterparts in higher eukaryotes [11].

In humans, the *Nse3* gene is represented by an expanded family of “*MAGE*” (melanoma antigen gene) genes with over 50 members, classified into two types. Type I MAGE genes are frequently over-expressed in human primary cancers and cancer cell lines, and may play a role in resistance to chemotherapeutic agents [18]. In fact, 85% of cancer cell lines over-express at least one Type I MAGE gene [19]. In contrast, Type II MAGE genes, such as *NDN*, *MAGEL2* and *MAGED1* are expressed in normal tissues and have important roles in mammalian development [20], [21], [22]. MAGEG1 was identified as a component of the human Smc5/6 complex [23]. The crystal structure of MAGEG1 revealed its interaction with RING protein Nse1, and this interaction stimulates the ubiquitin ligase activity of Nse1 [17], [23]. Other MAGE proteins interact with the mammalian homologs of Nse1 and Nse4, suggesting a conserved role of MAGE proteins as part of distinct Smc5/6 complexes [15], [17], [23], [24], [25].

All components of the Smc5/6 complex are essential in *S. cerevisiae* [13], and, except for Nse5 and Nse6, also in *S. pombe* [11]. Many hypomorphic *Smc5/6* mutants are hypersensitive to genotoxic agents such as ionizing radiation (IR), the alkylating agent methyl methanesulfonate (MMS), hydroxyurea (HU) and UV light in yeasts [26]. Epistasis experiments in yeasts and vertebrate cells have placed *Smc5/6* genes in the homologous recombination-based DNA repair pathway that involves Rad51 nucleofilament proteins [8]. In *Drosophila*, Smc5/6 plays a role in maintaining genome stability in heterochromatin regions by repressing non-sister chromosome recombination events [9], [27]. *Drosophila* Smc5/6 also serves a conserved molecular role in blocking Rad51 loading during this process and compromising Smc6 activity in S2 cells caused chromosome defects, suggesting Smc5/6 functions are essential [27]. Regulation of homologous recombination-mediated repair relies largely on two kinases, ataxia telangiectasia mutated (ATM) and ataxia telangiectasia and Rad3 related (ATR). ATM and ATR are phosphoinositide 3-kinase-like kinases (PIKK) that are activated by double strand breaks, turning on a network of DNA damage response signaling pathways that coordinate cell cycle progression and DNA repair [28]. Caffeine is a PIKK inhibitor commonly used to inhibit ATM and ATR [29], [30]. We sought to identify novel genes functioning in DNA damage response pathways that are redundant with ATM and ATR, by screening for conditional eye phenotypes in adult flies that were fed caffeine throughout larval development. We found unexpectedly that three *Drosophila* genes, *Smc5*, *Smc6* and *MAGE*, are not essential under normal growth conditions, but are essential for resistance to caffeine exposure throughout development. Interestingly, these mutants are also hypersensitive to genotoxic agents, suggesting a conserved role for the Smc5/6 in DNA damage repair. Caffeine induces apoptosis in the mutant flies in a process mediated by ATM and ATR that does not involve conventional cell cycle checkpoints. We have thus identified a novel caffeine-sensitive mechanism that prevents apoptosis in cells exposed to genotoxic stress.

## Results

### A Screen for Caffeine-sensitive Eye Mutants Reveals Three Loci on Chromosome 3R

**Figure 1:**
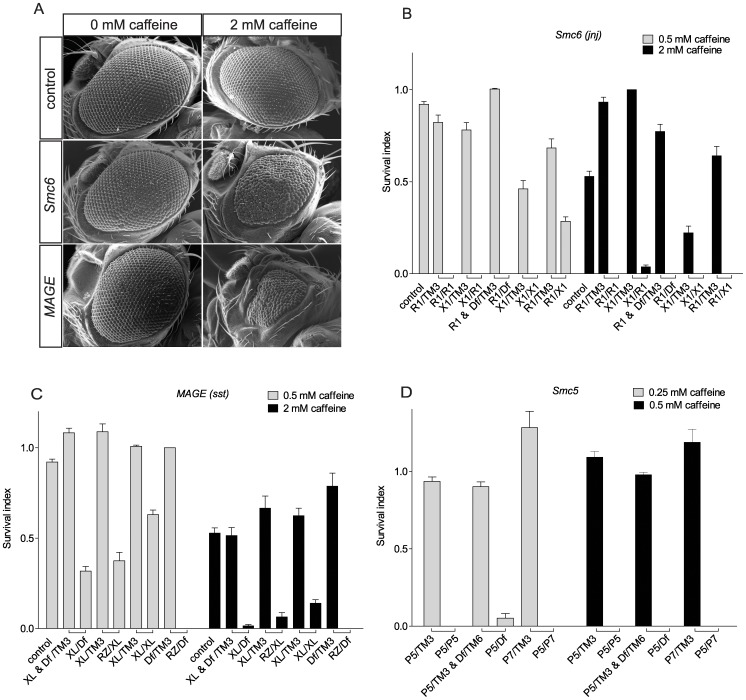
Eye phenotypes in caffeine-sensitive mutant flies. (A) Caffeine-dependent eye phenotype of *Smc6 (jnj)* and *MAGE (sst)* mutants. Fly genotypes are as follows. Control: *EGUF/+; FRT82B +/FRT82B GMR-hid*. *Smc6* (loss of *Smc6* in eye cells): *EGUF/+; FRT82B jnj^R1^/FRT82B GMR-hid. MAGE* (loss of *MAGE* in eye cells): *EGUF/+; FRT82B sst^RZ^/FRT82B GMR-hid*. (B-D) *Smc6, MAGE* or *Smc5* homozygous, trans-heterozygous or hemizygous mutants have reduced survival when raised in media with caffeine. Bars represent the survival index (*p*) and error bars represent SEM. “□” indicates flies eclosed from the same cross. Absence of a bar indicates no surviving flies. Wildtype control flies are *w^1118^*. (B) *Smc6* mutants are sensitive to caffeine. *R1* (*jnj^R1^*) is an allele from the caffeine screen, *X1* (*jnj^X1^*) was generated by an imprecise excision of a P-element adjacent to the 5′UTR of *Smc6*, and *Df* (*Df(3R)Exel6198*) is a deficiency chromosome uncovering the *Smc6* locus. (C) *MAGE* mutants are sensitive to caffeine. *RZ* (*sst^RZ^*) is an allele from the caffeine screen, *XL* (*sst^XL^*) is a targeted knockout, and *Df* (*Df(3R)Antp^1^*) is a deficiency chromosome uncovering the *MAGE* locus. (D) *Smc5* mutants are sensitive to caffeine. Both *P5* (*Smc5^P{GSV1}GS3245^*) and *P7* (*Smc5^P{GSV6}GS14577^*) contain P-element insertions in a coding exon of *Smc5*, and *Df* (*Df(3L)BSC418*) is a deficiency chromosome uncovering the *Smc5* locus.

The compound eyes of *Drosophila* are ideal tissues to detect defects in proliferation and apoptosis as they are not essential for survival, but they are sensitive to developmental perturbations and easy to score for mutant phenotypes. To identify novel genes functioning in DNA damage response pathways that are redundant with ATM and ATR, we previously performed a genetic screen to identify conditional eye phenotypes in adult flies fed 2 mM caffeine and 3 mM hydroxyurea (HU) throughout larval development [31]. While caffeine inhibits ATM and ATR, HU stalls replication forks through inhibition of dNTP production, eventually generating single strand or double strand DNA breaks, thereby activating DNA damage responses regulated by ATM and ATR. At the drug concentrations used, there were no phenotypic effects in wildtype flies. In this screen, we used the “*EGUF, GMR-hid*” (*EGUF*) system to produce homozygous mutant clonal cells in the entire adult eye of an otherwise heterozygous fly [32]. This screen identified a single caffeine-sensitive locus (*huc95E*) on chromosome arm 3R, here renamed *java no jive* (*jnj*), which we mapped to cytological region 95E by complementation testing with chromosomal deficiencies [31]. Flies that were mosaic hemizygous for *jnj* in the eye exhibit caffeine-dependent small, rough eyes associated with increased apoptosis. To identify novel DNA damage pathway components, we have now carried out a new screen of chromosome arm 3R for conditional caffeine-sensitive eye phenotypes. By screening 9098 males, we identified three loci on chromosome arm 3R including six additional alleles of *jnj*, two mutant alleles of a locus called *sleepless in seattle* (*sst*), and one allele of a novel locus called *double double trouble* (*ddt*), that has not yet been linked to a specific gene (Fig. 1A, Fig. S1). All hemizygous *jnj, sst* and *ddt* mutants exhibit caffeine-dependent pupal lethality (Fig. 1B–D and data not shown).

### Mutations in *Smc6* Cause Caffeine-dependent Defects in *java no jive* Mutant Flies

**Figure 2:**
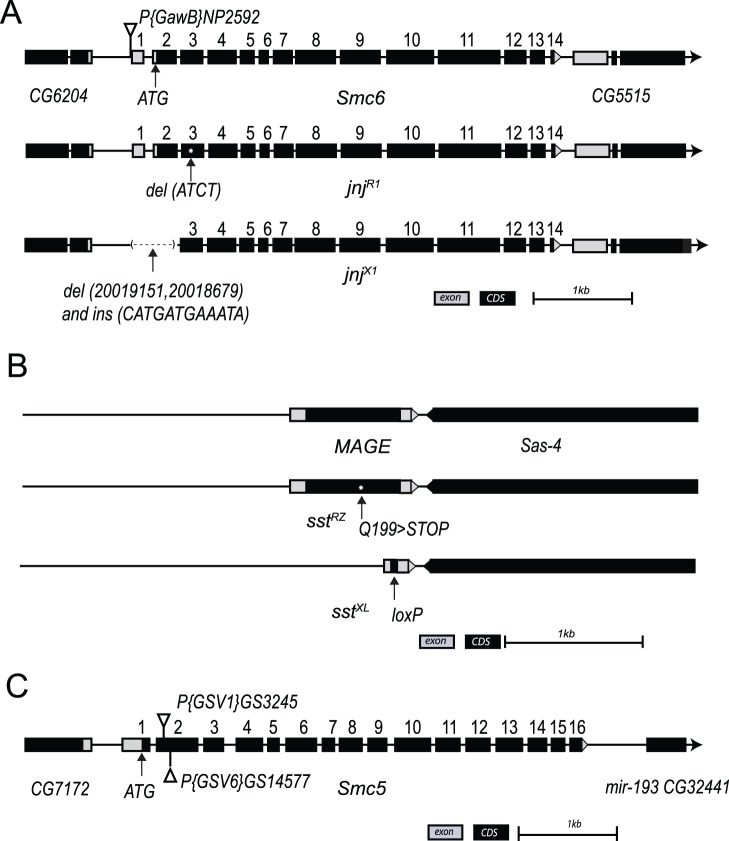
Overview of *Smc6, MAGE*, and *Smc5* gene location, structural organization and mutant alleles. (A) *Smc6* is a 14 exon gene located on 3R:95E8–95F1. *jnj^R1^* contains a 4 bp deletion in the 2nd coding exon. *jnj^X1^* contains a 473 bp deletion of sequences upstream of exon 1 (196 bp), the entire exon 1 (252 bp), and a portion of intron 1 (25 bp), with a 12 bp vestige of the original P element remaining. *Smc6* genomic locus (3R:20,014,770.20,019,145 [−]) is shown. (B) *MAGE* is a single exon gene located on the right arm of the 3rd chromosome at position 84C7–84C7. *sst^RZ^* has a point mutation that converts a glutamine at position 109 to a stop codon. *sst^XL^* carries a targeted deletion of the entire coding sequence of *MAGE*. *MAGE* genomic locus (3R:2,979,960.2,980,898 [−]) is shown. (C) *Smc5* is a 16 exon gene located in 78D6–78D7 of the left arm of the 3rd chromosome. Exons encoding the longest transcripts are shown. Both *P{GSV1}GS3245* and *P{GSV6}GS14577* are inserted in the second coding exon. The *Smc5* genomic locus (3L:21,562,309.21,566,623 [+]) is shown. CDS, coding sequence.

Deletion mapping indicated that all of the caffeine-sensitive *jnj* alleles were viable in hemizygous combinations with deletions uncovering region 95E, indicating that the homozygous lethality of most *jnj* alleles was caused by second site mutation(s). Homozygotes for one allele, *jnj^R1^*, were viable on regular media, but died at the pupal stage when raised in media containing caffeine (Fig. 1B). Sequencing of candidate genes in the *jnj* region identified a four base pair deletion in exon two of the FlyBase annotated gene *CG5524* (del_ATCT at position 334–337 bp from the presumptive start codon), creating a frameshift resulting in a stop codon at position 133 of the presumptive 1122 amino acid protein (Fig. 2A). The predicted CG5524 protein has highest amino acid identity with SMC6 (Structural Maintenance of Chromosomes 6) in other species. SMC6 regulates chromosome stability in yeasts [7], [8], [9], and is implicated in heterochromatic DNA repair in *Drosophila* [27]. We tested *CG5524* (hereafter called *Smc6*) and four neighboring genes for levels of expression by quantitative RT-PCR of RNA from whole flies. Levels of *Smc6* RNA were greatly reduced with all seven alleles of *jnj*, ranging from 9% to 24% of control levels (Fig. S2A) whereas nearby genes showed little change in expression. Despite extensive sequencing efforts, we were not able to identify the nature of *jnj* alleles other than *jnj^R1^*, suggesting that these unmapped mutations reside in as yet unidentified regulatory regions of *Smc6*. To be certain that our *jnj* alleles corresponded to *Smc6*, we generated additional *Smc6* lines by imprecise excision of the P-element present in line *NP2592*, including the new line *jnj^X1^* that lacks exon 1 and sequences up- and downstream of this exon (Fig. 2A). We tested caffeine sensitivity in all of the *jnj* allelic combinations and found that raising larvae on 0.5 mM caffeine resulted in almost complete lethality (Fig. 1B). Using RNAi to deplete *Smc6* expression in developing eye discs also resulted in a caffeine-dependent rough eye phenotype (Fig. S2B). Collectively, the presence of a frame shift mutation in *Smc6* in *jnj^R1^*, the reduced expression levels of *Smc6* in all seven alleles of *jnj*, the caffeine-dependent lethality of the deletion allele *jnj^X1^*, and caffeine-dependent eye phenotypes induced by *Smc6* RNAi all implicate *CG5524/Smc6* as the relevant gene in *jnj* mutants.

### Caffeine-sensitivity in *sleepless in seattle* Mutants is Due to Mutations in the *MAGE* Gene

The *sst^RZ^* mutation exhibits caffeine-dependent pupal lethality in combination with a chromosomal deficiency (*Df(3R)Antp^1^*, Fig. 1C) but *sst^RZ^* homozygotes are not viable on regular media, presumably because of a second site mutation. Further deletion mapping refined the position of the caffeine-sensitive *sst* locus to a region containing seven candidate genes, each of which were sequenced. We identified a glutamine to stop mutation affecting the *MAGE* gene [33] in *sst^RZ^*, at position 109 of the 232 amino acid Mage protein (Fig. 2B). In previous studies, depletion of *MAGE* mRNA using double strand RNA injection suggested that *MAGE* was essential for viability during early embryogenesis, whereas conditional knockdown at later developmental stages suggested a role in postembryonic neuronal survival and proliferation [34]. Moreover, DNA fibers connecting mitotic cells were observed after RNAi-mediated depletion of *Smc5* or *Smc6* in *S2* cells, suggesting that the Smc5/6 complex could be essential for mitosis in *Drosophila* [27]. We therefore initially reasoned that *sst^RZ^* was a partial loss-of-function allele, since hemizygous *sst^RZ^* flies were viable. To test this idea we synthesized a knockout allele by homologous recombination [35]. In this new allele (*sst^XL^*) the complete coding sequence of *MAGE* was deleted (Fig. 2B). Surprisingly, homozygous *sst^XL^* flies displayed no increased lethality or obvious mutant phenotype when raised on media without caffeine. As with *sst^RZ^* hemizygotes, *sst^XL^* flies reared in caffeine media were inviable, but they were less sensitive to a lower dose of caffeine (0.5 mM) than *jnj* mutants (Fig. 1C). About 15% of predicted *sst^XL^* homozygous flies survived 2 mM caffeine exposure and the surviving flies often had small or rough eyes, similar to *sst^RZ^* mutants (Fig. 1A). Transheterozygous *sst^RZ^*/*sst^XL^* progeny were also viable on normal media, but only 6% survived on 2 mM caffeine (Fig. 1C). Using polyclonal antibodies directed against Mage [36] we found that Mage was absent from protein lysates derived from *sst* adult flies (Fig. S3). In addition, caffeine-dependent lethality of *sst^XL^* can be complemented by a genomic *MAGE* transgene (Table S1) that includes the full coding region of *MAGE* and 3 kb sequence upstream and expresses Mage protein at normal levels (Fig. S3). Collectively, the identification of a stop mutation in the *MAGE* gene (*sst^RZ^*), the caffeine-sensitivity of a *MAGE* knockout allele *sst^XL^*, the loss of Mage protein in *sst* flies and the rescue of caffeine sensitivity by a *MAGE* transgene all implicate *MAGE* as the mutated gene in *sst* flies.

### *Smc5* Mutant Flies are also Caffeine Sensitive

**Figure 3:**
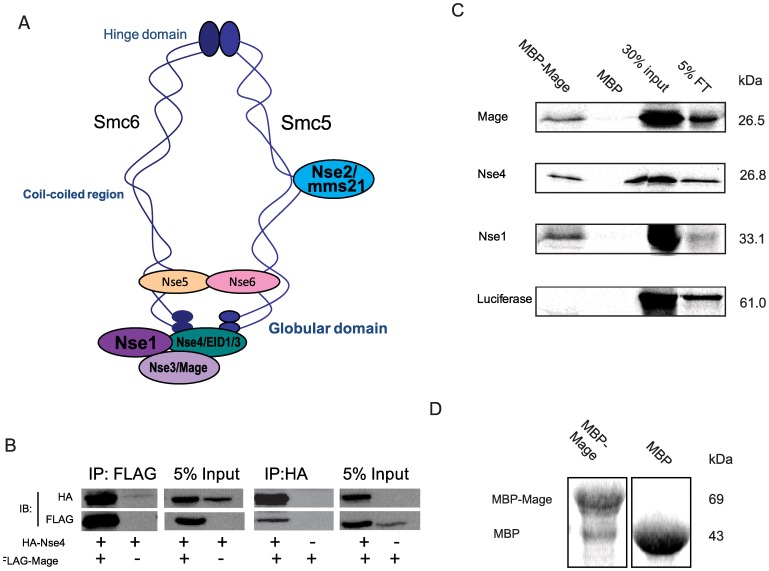
Mage is part of the *Drosophila* Smc5/6 complex. (A) Diagram of a generic Smc5/6 complex in *S. pombe* (adapted from [70]). The structure in *S. cerevisiae* is different in that Nse5/6 were found to bind at the hinge. (B) Mage interacts with Nse4 when both proteins are co-expressed in *S2* cells. HA-Nse4 co-immunoprecipitated (co-IP) with FLAG-Mage from an *S2* cell lysate when two proteins were co-expressed; FLAG-Mage co-IPed with HA-Nse4 from the *S2* cell lysate when two proteins were co-expressed. (C) Recombinant Mage interacts with Nse4 and Nse1 directly. Immobilized maltose binding protein (MBP)-fused MAGE or MBP were incubated with ^35^S-methionine labeled Mage, Nse4, Nse1, or luciferase (as a negative control), respectively. Proteins that were associated with immobilized MBP-Mage or MBP were resolved with SDS-PAGE and visualized by autoradiography. Results show that Mage, Nse4, and Nse1 each interact with MBP-Mage but not with MBP and luciferase does not interact with either of these proteins. (D) Coomassie staining of protein immobilized on 10 µl of amylose beads showed that approximately equal amounts of MBP-Mage and MBP proteins were immobilized on resin beads.

In yeasts and mammalian cells, all known SMC6 functions involve SMC5 [23], [37], so we predicted that loss of *Smc*5 activity would also cause caffeine sensitivity in flies (Fig. 3A). We tested two P insertion alleles predicted to affect *Smc5* for caffeine sensitivity, namely *Smc5^P{GSV1}GS3245^*, referred to as *Smc5^P5^*, and *Smc5^P{GSV6}GS14577^*, referred to as *Smc5^P7^* [38]. As predicted, both *Smc5* mutants were sensitive to caffeine (Fig. 1D). Both of these alleles have P-element insertions within the second exon of *Smc5* and the insertion sites are very close to the putative start codon (Fig. 2C). Therefore, they are very likely to be null alleles. To rule out the possibility that caffeine-sensitivity of *Smc5* flies was caused by second site mutations, we generated fly lines in which the P-elements in both alleles were excised by a transposase, either restoring the wild-type sequence or resulting in an insertion or deletion of the original P element insertion in the coding exon of *Smc5*. We therefore predicted that some excision lines would no longer be caffeine-sensitive while others would retain the mutant phenotype. As expected, of 13 independent fly lines produced by the excision of *P7*, seven lines were no longer caffeine sensitive (Table S2A). Similar results were obtained from the excision of *P5* (Table S2B). In conclusion, as with *Smc6* and *MAGE*, loss of *Smc5* function results in caffeine-dependent lethality.

### Caffeine Sensitivity is Mediated through Smc5/6

At the whole organism level, a higher proportion of *MAGE* mutants were able to survive exposure to 0.5 mM caffeine throughout larval development than *Smc6* and *Smc5* mutants. Indeed all genetic combinations of *MAGE* mutant flies had some survivors on media containing 2 mM caffeine, while there were essentially no survivors among the *Smc5* or *Smc6* mutants raised on 2 mM caffeine (Fig. 1B–D). This suggests that the Mage protein is less important for caffeine resistance than the Smc5 and Smc6 proteins. To further test this hypothesis, we measured the viability of flies carrying mutations in two different components of the protein complex when raised on media containing caffeine. Flies deficient for both Mage and Smc6 were more sensitive to caffeine than flies deficient for Mage alone, but were similar in sensitivity to flies deficient for Smc6 alone (Table S3). This suggests that the Smc5/6 heterodimer has a more critical role in caffeine resistance than does the sub-complex containing Nse1-Mage, consistent with observations in yeasts [1].

### *Drosophila* Smc5/6 Components Form a Protein Complex

In yeasts, the Smc5/6 complex consists of Smc5, Smc6 and six Nse (non-Smc element) subunits [14], four of which were also identified in humans [15], [23]. In searches of *Drosophila* genome databases, we uncovered a set of putative transcription units that appear to correspond to SMC5/6 complex subunits in yeasts (Table S4). Of these, *MAGE* has previously been described as a homolog of yeast *Nse3* and human *MAGEG1* [23]. In *Drosophila*, Mage protein was shown to interact with *Drosophila* Nse4 (Nse4) using a yeast two-hybrid system [39]. When we examined the Gene Expression Omnibus (GEO, [40]) to compare gene expression profiles, we found that these two genes have very similar expression patterns across different tissues, supporting the idea that the encoded proteins function in a complex (Fig. S4). Fission yeast Nse1 has been detected in the same sub-complex as Nse3 and Nse4, as part of the larger Smc5/6 complex (Fig. 3A) [11]. We first tested for a physical interaction between *Drosophila* Mage and Nse4 in cell culture, by generating epitope-tagged plasmid constructs that produce HA-tagged Nse4 or FLAG-tagged Mage, and co-transfecting them into *Drosophila* Schneider 2 (S2) cells. We were able to co-immunoprecipitate HA-Nse4 and FLAG-Mage from S2 cell lysates (Fig. 3B). We then performed *in vitro* pull down experiments to show that this interaction is likely direct, and that Mage also interacts with Nse1 directly (Fig. 3C). These results indicate that the three *Drosophila* proteins (Nse1, Mage and Nse4) form a sub-complex analogous to that found in yeast, consistent with conservation of structure across species.

### Loss of Function for *Smc6* or *MAGE* Sensitizes Imaginal Cells to Caffeine-induced Apoptosis

**Figure 4:**
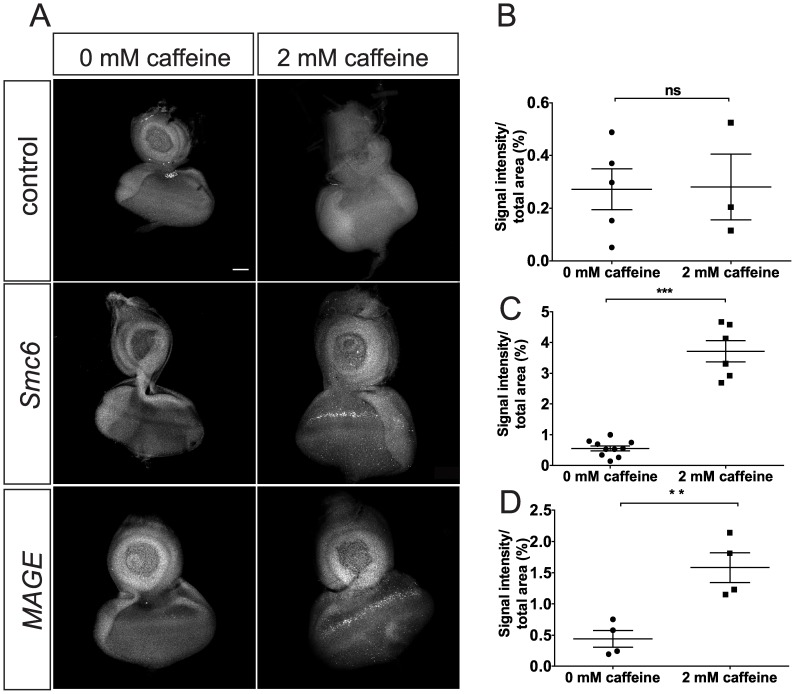
Caffeine exposure results in apoptosis in eye discs of *MAGE* and *Smc6* mutants. (A) Anti-cleaved-caspase-3 antibody staining of eye discs from third instar larvae of control (*WT*, *FRT82B*), *MAGE* (*sst^RZ^*/*sst^XL^*), and *Smc6* (*jnj^X1^/jnj^R1^*) genotypes raised in either standard media (0 mM caffeine) or media supplemented with 2 mM caffeine for 12 hours before dissection. Images are single stacks of confocal images. More cleaved-caspase-3 foci in eye discs of *sst^RZ^*/*sst^XL^* and *jnj^X1^/jnj^R1^* larvae were observed after caffeine exposure. A narrow band of apoptotic cells (white arrow heads) anterior to the presumptive morphogenetic furrow are most noticeable. Scale bar represents 50 µM. (B-D) Quantification and comparison of cleaved caspase-3 staining levels in *WT* (B), *MAGE* (C) or *Smc6* (D) eye discs, comparing the no caffeine and 2 mM caffeine groups. Data represent mean area stained from multiple eye discs for each genotype per treatment. A maximum projection of all stacks of a confocal image was used to quantify the signal intensity of staining. This value was divided by the area of each eye disc to obtain a ratio representing the relative amount of immunostaining. Error bars represent SEM. A non-paired two-tailed *t*-test was used to determine statistical significance. **, *P* = 0.006, ***, *P*<0.0001.

Previous examinations of *jnj^huc95E^* hemizygous mutants were based on the EGUF eye mosaic system [31]. In this experiment, we observed caffeine-dependent defects in ommatidial patterning and increased apoptosis in the eye discs. Larvae mutant for *Smc6* or *MAGE* die at the pupal stage when raised long term on caffeine-containing media. Remarkably, upon dissection of these larvae we noticed that the imaginal discs were severely damaged or altogether absent, suggesting increased cell death as the cause of this defect. To test this hypothesis, we dissected eye imaginal discs from late third instar larvae and labeled them with antibodies against activated caspase 3 to mark apoptotic cells. We detected minimal labeling of apoptotic foci in eye discs of control larvae, regardless of caffeine exposure (Fig. 4). In contrast, dramatically increased labeling of apoptotic foci were seen in the eye discs of *Smc6* or *MAGE* mutant third instar larvae after short term (12 hours) caffeine exposure. Apoptotic labeling was markedly enhanced in a band of cells immediately anterior to the morphogenetic furrow, where cells become synchronized in G1 phase [41]. These results suggest that caffeine-induced apoptosis in developing imaginal discs likely underlies caffeine-dependent pupal lethality in *MAGE* and *Smc6* mutant flies.

### *Smc5/6* Mutant Flies are Hypersensitive to Genotoxic Stress

**Figure 5:**
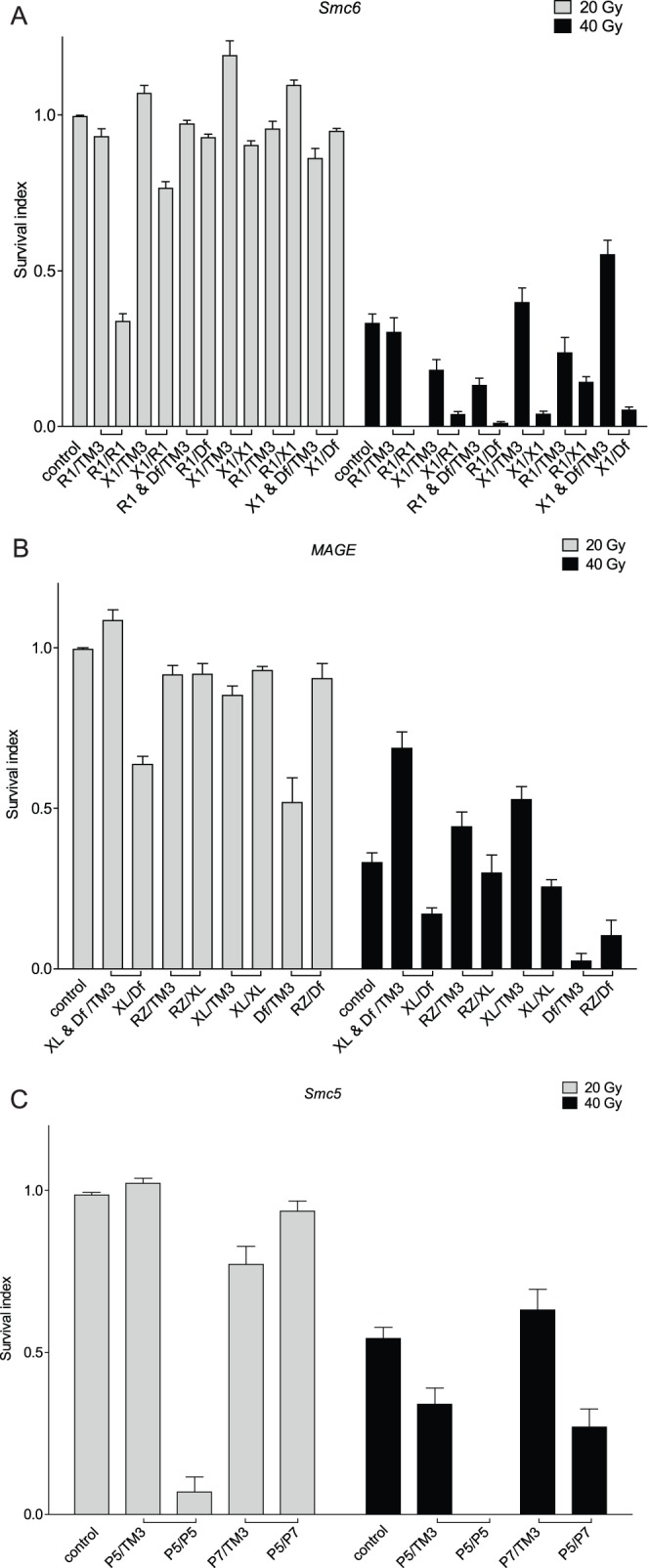
*Smc5/6* mutants are hypersensitive to ionizing radiation. (A–C) *Smc6, MAGE* or *Smc5* homozygous, trans-heterozygous or hemizygous mutants have reduced survival when exposed to 40 Gy of IR. Bars represent the survival index (*p*) ± SEM. “□” indicates flies eclosed from the same cross. Absence of a bar indicates that no flies survived at that IR dose. (A) *Smc6* mutants are hypersensitive to IR. *R1* (*jnj^R1^*) and *X1* (*jnj^X1^*) are *Smc6* alleles. *Df* (*Df(3R)Exel6198*) is a deficiency chromosome uncovering the *Smc6* locus. (B) *MAGE* mutants are hypersensitive to IR. *RZ* (*sst^RZ^*) and *XL* (*sst^XL^*) are *MAGE* alleles. *Df* (*Df(3R)Antp^1^*) is a deficiency chromosome uncovering the *MAGE* locus. (C) *Smc5* mutants are hypersensitive to IR. *P5* (*Smc5^P{GSV1}GS3245^*) and *P7* (*Smc5^P{GSV6}GS14577^*) are *Smc5* alleles. *Df* (*Df(3L)BSC418*) is a deficiency chromosome uncovering the *Smc5* locus.

The DNA damage response is a multi-step process that involves sensing of damage, cell cycle arrest, and repair of the damaged DNA. Yeast with hypomorphic mutations affecting *Smc6*, *Nse1*, *Nse2*, *Nse3* or *Nse4* are hypersensitive to gamma irradiation, UV light, MMS, camptothecin (a topoisomerase I inhibitor), and inhibition of DNA replication by HU [26]. All of these genotoxic stresses directly or indirectly generate DNA single-stranded or double-stranded breaks. To explore whether *Drosophila* Smc5/6 provides similar responses to genotoxic stress, we analyzed the effects of ionizing radiation, camptothecin, HU or MMS on viability. Exposure to 40 Gy ionizing radiation caused increased lethality in *MAGE*, *Smc6* and *Smc5* mutants compared to controls (Fig. 5). Moreover, all three mutants were hypersensitive to camptothecin, HU and MMS, compared to controls (Figs. S5 and data not shown).

### Loss of *Smc5/6* Function does not Compromise G2/M and S Phase Checkpoints Induced by Genotoxic Agents

**Figure 6:**
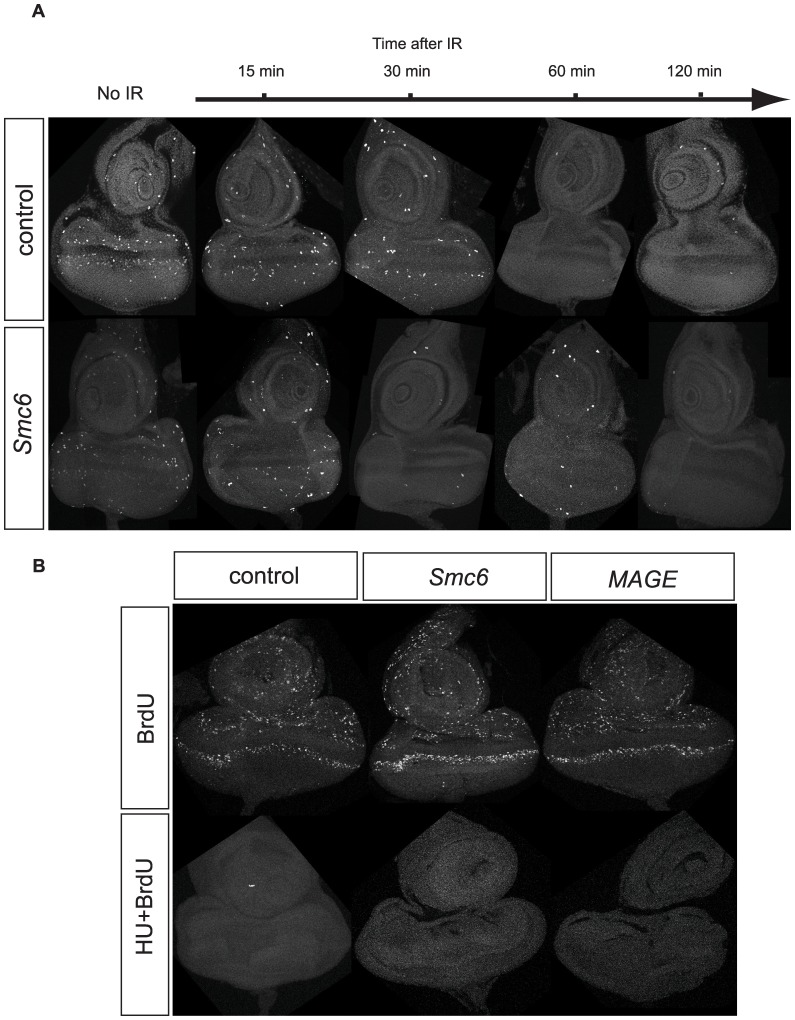
*Smc5/6* genes are not required for G2/M and S phase checkpoints induced by genotoxic agents. (A) Wandering third instar larvae were irradiated with 40 Gy of ionizing radiation and the eye-antenna discs were dissected and fixed 15 minutes, 30 minutes, 1 hour or two hours after radiation, with discs from unirradiated larvae serving as controls. Representative images of PH3 staining for mitotic cells in eye-antenna discs from control (*WT, FRT82B*) and *Smc6*, (*jnj^R1^/jnj^X1^*) transheterozygous larvae are shown. (B) Eye-antenna discs from wandering third instar larvae were incubated with or without HU before adding BrdU to the incubation solution. Representative images of BrdU staining for cells in S phase in eye-antenna discs from control (*WT, FRT82B*), transheterozygous *Smc6* (*jnj^R1^/jnj^X1^*) or transheterozygous *MAGE* (s*st^RZ^/sst^XL^*) eye-antenna discs are shown.

Studies in *Drosophila* have proven to be valuable for the study of proteins and pathways controlling DNA repair and checkpoint responses, which are remarkably well conserved among flies and other organisms [27], [42]. In *S. pombe*, *nse3-1* hypomorphic mutants activate a DNA damage checkpoint that arrests cells in late S phase/G2 [7], and *Smc6 (Rad18)* is required for maintenance but not activation of the G2 checkpoint [43], [44]. We therefore tested whether cell cycle checkpoints important for DNA damage response pathways were perturbed in caffeine-sensitive *MAGE* or *Smc6* mutant flies. To assess G2/M checkpoint function we used ionizing radiation (IR) to determine if IR exposure decreased the number of mitotic cells [45]. We dissected eye imaginal discs from late third instar larvae and labeled them with anti-phospho histone H3 antibodies to mark mitotic cells. The number of mitotic cells in un-irradiated eye imaginal discs of *jnj^R1^ (Smc6)* or *sst^XL^ (MAGE)* larvae was comparable to that of control eye discs (Fig. 6A). Larvae were exposed to 40 Gy of IR and dissected eye discs were examined from 15 to 120 min. after exposure. Phospho-histone H3 foci disappeared after 30 or 60 min in wild-type (*Iso)* controls, *jnj^R1/X1^ (Smc6)* and *sst^XL/RZ^ (MAGE)* eye discs (Fig. 6A), demonstrating that neither Mage nor Smc6 is required for activation of the G2/M checkpoint.

The caffeine sensitive ATM/ATR kinases are important mediators of DNA damage checkpoints [28]. In *S. pombe*, the SMC5/6 complex is recruited to and stabilizes stalled replication forks after Rad3 (ATR homolog) activation [46]. To investigate whether the S phase checkpoint was intact in *jnj^R1/X1^ (Smc6)* and *sst^XL/RZ^ (MAGE)* mutant flies, we monitored BrdU incorporation pattern in eye imaginal discs before and after treatment with HU, which induces the S phase checkpoint [47]. We observed many S-phase cells incorporating BrdU in control untreated eye discs, however incorporation was abolished upon exposure to HU. BrdU incorporation was also abolished by HU treatment in *jnj^R1/X1^* and *sst^XL/RZ^* mutant discs (Fig. 6B), demonstrating that Mage and Smc6 are also not essential for S phase checkpoint activity in *Drosophila*.

### *Smc6* and *MAGE* Genetically Interact with Proteins Required for DNA Damage Responses

**Figure 7:**
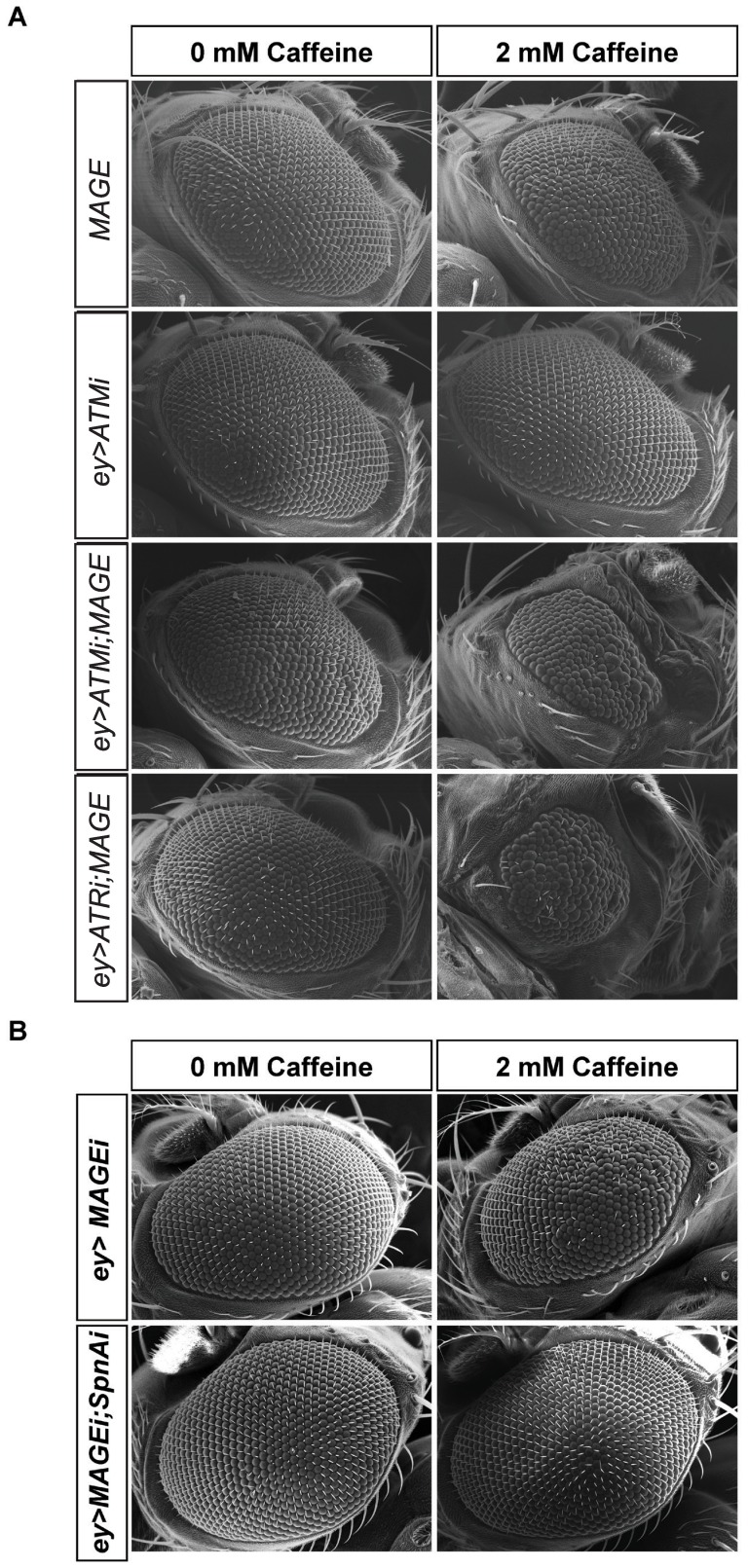
Caffeine-dependent genetic interaction of *MAGE* with *ATM*, *ATR* and *Rad51*(*SpnA*). (A) Representative eye phenotypes of *MAGE* (*EGUF/+; FRT82B sst^RZ^/FRT82B GMR-hid*, loss of *MAGE* in eye cells), *ey>ATMi* (knockdown of *ATM* in eye cells), *ey>ATMi;MAGE* (*EGUF/UAS-ATM-RNAi;FRT82B sst^RZ^/FRT82B GMR-hid,* loss of *MAGE* and knockdown of ATM in eye cells) and *ey>ATRi;MAGE* (*EGUF/UAS-ATR-RNAi;FRT82B sst^RZ^/FRT82B GMR-hid,* loss of *MAGE* and knockdown of ATR in eye cells) flies that were reared on either standard media or media containing 2 mM caffeine. The EGUF system carrying the *eyeless-Gal4* driver was used to drive the UAS-RNAi transgenes in the eye and also makes the eye homozygous for *MAGE* (*sst^RZ^)*. Controls for the effects of each eyeless-driven RNAi alone were carried out for *ATM* and *ATR* resulting in wild type appearing eyes, but only the results of *ATM* RNAi are shown here as an example. (B) Representative eye phenotypes of *MAGE* knockdown (*eyeless-Gal4/+;UAS-MAGE-RNAi/UAS-Dicer2,* knockdown of *MAGE* in eye cells) and *MAGE Rad51* double knockdown (*eyeless-Gal4/UAS-SpnA-RNAi;UAS-MAGE-RNAi/UAS-Dicer2*, knockdown of *MAGE* and *Rad51* in eye cells) flies that were reared on either standard media or media containing 2 mM caffeine.

Caffeine inhibits ATR and ATM kinase activity [29], [30], raising the possibility that partial loss of ATM or ATR function could be contributing to the caffeine-induced defects that we observed in *Smc5/6* mutant flies. We therefore examined whether genetically reducing ATM or ATR function in an *Smc6* mutant background would cause synthetic lethality. The *Drosophila* homolog of ATR is Mei-41 [48] and *mei-41* mutants are homozygous viable but not caffeine-sensitive on their own [31]. To test for genetic interactions between *mei-41* and *Smc6*, we generated double mutants and measured the proportion that survived to adulthood when raised on caffeine-free media. There was no increased lethality associated with *mei-41;Smc6* double mutants (Table S5), implying that the inhibition of ATR alone by caffeine was not the main cause of caffeine-dependent lethality of *Smc6* homozygotes. To further examine genetic interactions between *ATR* and *MAGE* or *Smc6*, we used the EGUF system as a more sensitive system for detecting mutant phenotypes than lethality. Raised on standard media, adult flies with homozygous *MAGE* mutant eyes were indistinguishable from control flies (Fig. 7). Raised on 2 mM caffeine, however, *MAGE* mutant eyes were moderately rough relative to control eyes. *ATR* RNAi alone caused no observable roughness in the eye but when *ATR* RNAi was expressed in MAGE-deficient eyes, moderate to severely rough caffeine-dependent eye defects were observed that were not seen on caffeine-free media (Fig. 7, quantification in Fig. S6). We then tested whether ATM plays a role in caffeine sensitivity. *Drosophila ATM* (*tefu*) null mutants are non-conditional pupal lethal [49], so we used the *EGUF* system to examine these interactions as well. *ATM*-RNAi knockdown alone produced a normal looking eye, either in the absence or presence of caffeine. When *MAGE* mutant eyes were combined with *ATM*-RNAi, however, we observed a range of caffeine-dependent rough eye phenotypes, similar to eye defects caused by *ATR*-RNAi in *MAGE*-deficient eyes (Fig. 7, S6). *ATR*-RNAi knockdown alone produced a normal looking eye, either in the absence or presence of caffeine. We noted differences in expressivity between the *MAGE-*deficient eyes (compare Figs. 1A and 7A) that could be caused by slight differences in the genetic background (the genetic interaction study used *CyO* balancers while the original screen had wild type chromosomes) or the accumulation of genetic modifiers. We propose that the caffeine-induced partial loss of function of both ATM and ATR causes the rough eye phenotype in the *MAGE*-deficient background, and that further loss of either ATM or ATR increases the severity of this phenotype, We also examined interactions with *NBS1*, a component of the MRN (Mre11, Rad50 and Nbs1) complex that collaborates with ATM in DNA repair and telomere maintenance [50]. While *NBS1*-knockdown alone produced no effect, a dramatic caffeine-dependent enhancement of the rough eye phenotype was observed when *NBS1*-RNAi was combined with eye-specific *MAGE* mutants (Fig. S7). These striking caffeine-dependent genetic interactions between *MAGE* and *ATR, ATM,* and *NBS1* suggest that these proteins act together in maintaining genome stability. Similar genetic interactions were observed between *ATR* and *ATM* in *Smc6* eye-specific mutants, supporting this conclusion (data not shown).

### *Drosophila MAGE RNAi* Caffeine Sensitive Phenotype is Rescued by Rad 51 Knockdown

In *Drosophila* and other organisms, Smc5/6 functions in the homologous recombination repair pathway in DNA double strand break repair [26], [51], [52]. Rad51 is a key component of the homologous recombination pathway, regulating the rate-limiting step of homology searching and strand invasion. In *Drosophila,* Smc5/6 prevents precocious Rad51 loading onto irradiation damaged heterochromatin region before it moves outside of the HP1a domain for proper repair [27]. In yeast, *Smc5/6* mutants accumulate unresolved DNA structures, and Smc5/6 actively resolves DNA mediated sister chromatin linkages [53], [54], [55]. We therefore tested whether the caffeine-dependent rough eye phenotype of *Smc5/6* mutants is related to deregulated Rad51 activity. Knocking down *Rad51* in the *MAGE*-RNAi background rescued the rough eye phenotype of *MAGE*-RNAi flies in 80% of the double RNAi flies raised on 2 mM caffeine (Figs. 7B, S8). Taken together, these data indicate that the caffeine sensitivity of the Smc5/6 complex or at least of *MAGE* mutants is largely attributable to improper Rad51 activity. It is also possible that Rad51 action is normal during HR, but the Smc5/6 complex mutants are unable to complete HR repair or resolve HR intermediates.

## Discussion

In a genetic screen for mutations conferring caffeine sensitivity in flies, we identified viable alleles of *Drosophila Smc6* (*jnj*; *CG5524*) and *MAGE* (*sst; CG10059*) as well as an unknown gene (*ddt*). Additional loss-of-function alleles created by imprecise P-element excision of *Smc6* (*jnj^X1^*) or targeted knockout of *MAGE* (*sst^XL^*) were also viable under normal conditions, but exhibited caffeine-sensitive lethality. Although no molecular lesions were identified for most *jnj* (*Smc6)* alleles, transcript levels were dramatically reduced in all these mutants when hemizygous, implying that either mutations in regulatory regions affected expression, or that, like *jnj^R1^*, transcripts were subjected to nonsense-mediated decay. There was no detectable *MAGE* expression in homozygous, transheterozygous, or hemizygous *sst* mutants. Furthermore, a genomic *MAGE* transgene restored expression and rescued the caffeine-dependent lethality of *sst* mutants. Loss of *Smc5* by P-element insertion also resulted in caffeine sensitivity. These genetic results as well as biochemical data showing physical interactions among SMC6, MAGE, Nse1 and Nse4 indicate that the *Drosophila* Smc5/6 complex is structurally and functionally conserved between yeast and flies. Our screen only covered one chromosome arm (3R) to obtain seven alleles of *Smc6* and two alleles of *MAGE*, representing ∼20% of the genome. Homologs of the remaining SMC5/6 components reside on chromosome arms 2L and 3L (Table S4) and were thus not discovered in our screen. As there are no known *Smc5/6* homologs mapping to the *ddt* locus, identifying this gene and screening remaining chromosome arms for mutations conferring caffeine sensitivity may lead to novel Smc5/6 components or other pathways in which *Smc5/6* is involved.

The SMC5/6 complex has been intensively studied in yeasts and human cells for its roles in chromosome replication, segregation and repair of DNA double strand breaks by homologous recombination [10]. Depletion of Smc5 or Smc6 in *Drosophila* tissue culture cells resulted in heterochromatin bridges in 50% of mitotic cells [27], suggesting that the *Smc5 or Smc6* genes would be essential for viability. On the contrary, we found that the loss of *Smc5, Smc6*, or *MAGE* did not result in lethality *in vivo*, and indeed homozygous mutant flies have been maintained for generations (data not shown). There was a slight reduction in hatching rates among null eggs from null mothers in some of the mutant lines, so we cannot rule out a contribution of the maternal RNA to viability in early development. We also did not observe DNA links between sister chromatids, excess aneuploidy, or translocations in mitotic chromosomes of neuroblast squashes from Smc5/6 mutant flies (data not shown). Homologs of *Smc5* and *Smc6* in *Caenorhabditis elegans* are also dispensable for viability, however the homozygous mutant strains were prone to sterility and germ cell defects because of compromised inter-sister chromatid recombinational repair and excessive germ cell apoptosis [56].

In both *S. cerevisiae* and *S. pombe*, genes encoding SMC5/6 and Nse1–4 are essential and hypomorphic mutants are sensitive to genotoxic agents [7]. In *C. elegans*, *smc-5* and *smc-6* mutant germ cells are also hypersensitive to IR and exhibit increased germ cell apoptosis even without IR exposure [56]. In vertebrates, *Smc5*-deficient chicken DT40 cells are sensitive to MMS and IR [52]. Interfering with the function of human *NSE2* by RNAi sensitizes HeLa cells to MMS-induced DNA damage [57]. The *Smc5, Smc6* and *MAGE* mutants described here are also sensitive to IR (40 Gy), HU (4 mM to 8 mM), camptothecin (0.025 mM) and MMS (0.05–0.015%), consistent with an evolutionarily conserved role in resistance to genotoxic agents. Components of the Smc5/6 complex may be responsible for existing *Drosophila* mutagen sensitive (*mus*) mutants (e.g. [58]) or may not yet be represented among these collections so constitute novel genes important for mutagen resistance.

Our experiments suggested that cells located just before the morphogenetic furrow in the imaginal eye discs of larvae lacking Smc5/6 components were most sensitive to caffeine (Fig. 4). Many of these cells normally become synchronized in G1 phase by being forced through mitosis through induction of the *Cdc25^stg^* gene suggesting that the *Smc5/6* and *MAGE* mutants described here are particularly sensitive to mitotic kinase Cdk1 activity when treated with caffeine [41]. G2/M checkpoint responses to DNA damage and the S-phase checkpoint induced by stalled replication forks were both intact in *Drosophila Smc6* or *MAGE* mutants, however. These results may be explained by accumulating evidence that yeast *Smc5/6* mutants undergo normal initiation of the checkpoint response but then fail to complete repair before entering mitosis leading to the formation of DNA bridges and aberrant mitosis [9], [43], [59], [60]. Consistent with this explanation, *Drosophila* MAGE and Smc6 mutants genetically interact with ATM and ATR to increase the severity of the caffeine-induced rough eye phenotypes (Fig. 7). Similar dependencies were also recently reported for *S. cerevisiae,* where *Nse2* mutants deficient in SUMO ligase activity were viable but needed Mec1 kinase (ATR) to survive, even in the absence of genotoxic stress [61].

Studies of protein complexes that are critical for cellular responses to genotoxic stress are also highly relevant to cancer therapy in humans. It is increasingly apparent that the gene expression signature of each tumor dictates in part the success or failure of chemotherapeutic treatment or radiotherapy [62]. The expression of human Type I MAGE genes is commonly dysregulated in cancer cells. Moreover, many studies have correlated the levels of expression of particular MAGE genes with therapeutic response, prognosis and probability of metastasis [18]. The unexpected synergy between caffeine and loss of SMC5/6 activity could potentially be exploited for new therapeutic strategies where one could preferentially sensitize checkpoint-compromised cancer cells to apoptosis. Although the therapeutic potential of caffeine for causing premature chromosome condensation in G1 checkpoint-compromised cancer cells has long been recognized, the concentrations needed to fully inhibit ATR kinases are toxic [63]. In cells exposed to UV-light, caffeine inhibits rescue of stalled replication forks by translesion DNA synthesis, causing a switch to homologous recombination that can result in chromosomal aberrations [64], [65]. Further studies are needed to elucidate the relationships among MAGE proteins, Smc5/6 components, and proteins such as ATM and ATR that are also important for resistance to genotoxic agents in normal and cancer cells. In turn, mechanistic understanding of how cells respond to genotoxic stress will aid in the selection and dose of chemotherapeutic agents that target specific disruptions to DNA damage response pathways, in order to improve cancer prognosis and survival.

## Materials and Methods

### *Drosophila* Stocks and Husbandry

All crosses were carried out at 25°C, and flies were maintained on media formulated at the Bloomington *Drosophila* Stock Center at Indiana University (BDSC) with *p*-Hydroxy-benzoic acid methyl ester or propionic acid as the fungicide. Stocks were obtained from the BDSC, the Vienna *Drosophila* RNAi Center (VDRC), or the *Drosophila* Genetic Resource Center at Kyoto (DGRC) or generated in our laboratories where specified. Fly stocks used were:

y^1^ w*; P{70FLP}11 P{70I-SceI}2B sna^Sco^/CyO, S2.

*w^1118^; P*{*70FLP*}10; *Sb* ^1^/*TM6*, *Ubx.*

y^1^ w^67c23^ P{Crey}1b; D*/TM3, Sb^1.^

*P{GawB}NP2592.*

w*; *Dr^1^/TMS, P{Delta2-3}99B.*

*P{GSV1}GS3245.*

*P{GSV6}GS14577.*

*P{ey3.5-GAL4.Exel}2.*

*C(1)DX, y *[*1*]* f *[*1*]*/w *[*1*]* mei-41[D3].*

*UAS-ATR-RNAi.*

*UAS-ATM-RNAi.*

*UAS-NBS1-RNAi.*

*UAS-SpnA-RNAi.*

*UAS-MAGE-RNAi/CyO (TRiP).*

### Ethyl Methanesulfonate (EMS) Screen for Caffeine-sensitive Mutants on Chromosome 3R

The isogenized fly stock *FRT82B* carries a transgenic Flippase Recognition Target (FRT) site inserted at polytene segment 82B on chromosome 3R and was used to screen for caffeine sensitivity. Adult male flies were mutagenized by feeding with 15 mM EMS dissolved in 1% sucrose for 12 h. After a one day recovery period, mutagenized males were crossed to *EGUF; FRT82B GMR-hid, CL/TM3, Sb* virgin females. Three to five F1 progeny *EGUF/+; FRT82B/FRT82B GMR-hid, CL* males with normal eye morphology were crossed to *EGUF; FRT82B GMR-hid, CL/TM3, Sb* virgin females. The F2 progeny were raised in media with 2 mM caffeine. Individual male non-balancer F2 flies displaying abnormal eye morphology in both eyes were backcrossed to *EGUF; FRT82B GMR-hid, CL/TM3, Sb* virgin females, and the F3 progeny were raised in media without caffeine to identify any flies with caffeine-independent eye defects. Once the caffeine-dependence of the eye phenotype was confirmed, each mutation was mapped by complementation with the original *jnj^huc95E^* allele [31] or using the *Drosophila* 3R deficiency kit (BDSC). Both the *jnj^R1^* and *sst^RZ^* lines emerged from this screen.

### Sequencing of Candidate Genes

Targeted re-sequencing of mapped caffeine-sensitive loci was used to identify mutations in candidate genes. Genomic DNA from 50 adult flies was extracted using DNAzol reagent (Invitrogen, Burlington, ON, Canada). Overlapping PCR fragments about 10 kb in size were amplified using a Long Range PCR kit (Invitrogen). These fragments covered each region predicted to contain a mutation and 10 kb on either side. The PCR products were sequenced using Illumina technology and data was analyzed with Bowtie software (Illumina Inc., San Diego, CA) [66]. Mutations were confirmed by Sanger sequencing with BigDye v3.1 (Applied Biosystems, Carlsbad, CA). Restriction digestion (BpmI) of a genomic PCR fragment was used to confirm the mutation in *jnj^R1^.*

### Generation of the *MAGE* Allele *sst^XL^* Using Gene Targeting

The “ends-out” method [35] was used to produce a targeted deletion of *MAGE.* Specifically, 3 kb genomic regions upstream and downstream of the *MAGE* genomic locus were amplified by PCR from a *Drosophila* BAC clone (BACPAC Resources Center, RP98-3E11), using the following PCR primers 5′-ATTCATGCGGCCGCCGAAACTCAAACGCAGCGAA and 5′-ATTCTAGGTACCGAGAAGTGCTAGCCATTTCGAG or 5′-ATTCTAGGCGCGCCGGAGTAAACGCGGAGTAGAATACC and 5′-ATTCATCGTACGGGAAGGGGATCAGGATTGAA. The two PCR fragments were subcloned into the NotI-KpnI (Acc65I) or AscI-BsiWI sites of the ends-out vector *P[w25.2]* to produce a donor construct *P[w25.2]_NK_AB*. Seven transgenic lines were generated by P element transformation of a *w^1118^* strain using *P[w25.2]_NK_AB* (BestGene Inc, Chino Hills. CA). The three lines in which the *P[w25.2]_NK_AB* was located on chromosome 2 were tested for efficient excision by crossing to a line carrying the *FLP* recombinase (*w^1118^; P{ry+t7.2 70FLP}10; Sb^1^/TM6, Ubx*). One of the three transgenic lines (*6030-1-6M*) with the highest excision efficiency was chosen as the donor line, and crossed to y^1^ w*; P{70FLP}11 P{70I-SceI}2B sna^Sco^/CyO, S2 (BDSC #6934). The parents were allowed to lay eggs for two days in a vial, and on the third day the larvae were heat-shocked for 1 h in a 38°C water bath. F1 virgin females were collected and crossed to *w^1118^*; *P*{*70FLP*}10; *Sb* ^1^/*TM6*, *Ubx* (BDSC #6938) males. About 100 F2 progeny were selected by screening for nonwhite flies from about 1000 independent crosses. Each of these progeny was crossed to *w* ^1118^; *P*{*70FLP*}10; *Sb* ^1^/*TM6*, *Ubx* to make stocks. Twenty five independent lines were identified that exhibited correct targeting as detected by PCR of genomic DNA and loss of Mage protein expression by immunoblotting with a guinea-pig anti-Mage antibody [36]. The *white* marker of these lines was removed by crossing to a line carrying a Cre recombinase (y^1^ w^67c23^ P{Crey}1b; D*/TM3, Sb^1^ (BDSC #851). The resulting lines were tested for heterozygote and homozygote viability under normal conditions, yielding the line named *sst^XL^*.

### Generation of a Genomic Rescue Construct for *MAGE* on Chromosome 2

Genomic DNA was isolated from the isogenized strain P{ry[+t7.2] = neoFRT}82B to PCR amplify (Sequal Prep Long PCR Kit, Invitrogen) a 4 kb fragment spanning from 3 kb upstream of the MAGE gene (genomic locus 3R:2983898, based on the predicted transcription start site), to 206 bp downstream MAGE stop codon (genomic locus 3R: 2979891). The PCR product was digested with the restriction enzyme XbaI and cloned into the pCasper-hs vector. Transgenic flies were generated by BestGene Inc.

### Generation of Additional *Smc6* Alleles by P-element Mediated Excision

The *Smc6* deletion allele *jnj^X1^* was generated by imprecise excision of a P element in *P{GawB}NP2592* (DGRC #104251). This insertion, hereafter referred to as *NP2592*, is located 7 bp upstream of the putative transcriptional initiation site of *CG5524* (*Smc6*) (3R:20,014,770.20,019,145). Its location was confirmed by genomic PCR using primers flanking the *NP2592* locus. To excise out *NP2592*, *NP2592* virgin females were crossed to w*; *Dr^1^/TMS, P{Delta2-3}99B* (BDSC #1610) males carrying a Δ2–3 transposase. Single virgin F1 females of genotype *ΔNP2592/TMS,{Δ2–3}99B* were crossed to *Ly/TM3, Sb* males. Single F2 males of genotype *ΔNP2592/TM3, Sb* were crossed to virgin *Ly/TM3, Sb* virgin females to establish balanced lines. About 200 candidate lines were produced and subsequently tested for sensitivity to 2 mM caffeine. Six lines were found to be homozygous viable but caffeine-dependent lethal. Genomic PCR was used to confirm that there were deletions around the original P insertion sites in these stocks. One of the resulting lines was renamed *jnj^X1^*.

### Molecular Characterization of *Smc5* Alleles

The location of *P{GSV1}GS3245* (BDSC #200582) and *P{GSV6}GS14577* (BDSC #205862) within coding exon 2 of the *Smc5* gene was confirmed by genomic PCR using primers 5′-CGTTTCCACGATTTGTTACTGACA and 5′-CGTTTTTGCTTCTTAACCAGATCAC. These lines were renamed *Smc5^P5^* and *Smc5^P7^*, respectively. *Df(3L)BSC418* (BDSC #24922) is a sequence mapped chromosome deletion (78C9;78E1) that includes the *Smc5* locus and nearby genes.

### Embryo Collection, Drug Administration and Ionizing Radiation (IR) Treatment

Parental flies were allowed to lay eggs in collection cages on apple juice agar plates with yeast paste for 20 h. The eggs were gently removed from the agar plates using distilled water and a brush and collected using a small cloth-bottomed basket, and then arrayed on new apple juice agar plates. For each drug or radiation treatment, at least 100 embryos were transferred with a thin layer of agar underneath into each of 3 vials containing medium. Drug stocks were pre-added into the media to the appropriate working concentration, with the exception of methyl methanesulfonate, which was added into the medium 48 hours after transferring the embryos. For drugs dissolved in DMSO, an equal amount of DMSO alone was added into medium fed to control flies. The following drugs were used: caffeine (Sigma-Aldrich, St. Louis, MO, stock 1 M in water, final concentration 0.25–2 mM); camptothecin (Sigma-Aldrich, stock 25 mM in DMSO, final concentration 0.025 mM), methyl methanesulfonate (Sigma-Aldrich, stock 99%, final concentration 0.005–0.015%) and HU (Sigma-Aldrich, stock 1 M, final concentration 4–8 mM). For IR, third instar larvae were irradiated at doses of 20 and 40 Gray using an irradiator (Gammacell 220–Cobalt-60, Atomic Energy of Canada, 1979). The survival index (*p*) of a given genotype was calculated by dividing the number of adult survivors of the genotype resulting from media with a given reagent concentration or treatment (*n*) by the number of adult survivors of the same genotype resulting from media without that reagent or treatment (*N*).

### Immunoblotting

For each sample, ten 3–4 day-old adult flies were collected, frozen in liquid nitrogen and ground using a pestle in a 1.5 ml eppendorf tube. Mild lysis buffer (50 mM Tris, 150 mM NaCl, and 1% Triton X-100, pH 8.0) was then added (10 µl per fly) to solubilize the tissue. The suspension was centrifuged at 20,000*g* for 10 min. at 4°C and the supernatant was mixed and boiled with 2X Laemmli Buffer. Proteins were resolved by SDS-PAGE and transferred onto PVDF membranes for immunoblotting. A 1∶2500 dilution of guinea pig anti-Mage serum was used to detect Mage protein [36].

### Genetic Interactions of *ATM*, *ATR, NBS1* and *RAD51* Loss-of-function with *MAGE* and *Smc6*

Double mutants of *ATR* and *Smc6* used *mei-41^D3^* [48] and *Smc6* alleles *jnj^X1^ and jnj^Df(3R)Exel6198^*. Knockdown of *ATM*, *ATR* or *NBS1* function in *MAGE* or *Smc6* homozygous mutant eye clones was achieved using the *EGUF* system, which uses the *eyeless-Gal4* driver to express transgenes throughout eye development [32]. The *EGUF* system also ensures that all ommatidia of the adult eye are homozygous for either *Smc6* or *MAGE* mutant alleles, because of an eye-specific *GMR-hid* transgene that eliminates non-mutant ommatidia. RNAi knockdown of *MAGE* alone or double RNAi of *MAGE* and *Rad51* ortholog *SpnA* in the eye was achieved by crossing appropriate RNAi constructs containing males to *UAS-Dcr2/CyO; ey-Gal4/TM3,Ser* virgin females. For each genotype, five to nine specimens were photographed, and representative phenotypes are shown.

### cDNA Clones, Cell Culture, Transfections, and Co-immunoprecipitation

Full-length cDNA clones for *Nse1* (GM14348) and *Nse4* (IP09347) were obtained from the Canadian *Drosophila* Microarray Centre, the *MAGE* (RE25453) clone was obtained from the *Drosophila* Genomics Resource Center (DGRC, Indiana University). *Drosophila* S2 cells (from the DGRC) were grown at 25°C in TNM-FH medium (SH30280.02, Thermo Scientific, Waltham, MA) supplemented with 10% fetal bovine serum. Expression constructs for transfection of S2 cells were created by inserting relevant full-length coding sequences into the *Drosophila* Gateway destination vectors (obtained from the DGRC). S2 cells were transfected with relevant expression constructs using dimethyldioctadecyl-ammonium [67]. Cells were harvested 24 h after transfection, washed once in phosphate buffered saline, pH 7.2, and re-suspended in the mild lysis buffer supplemented with a protease inhibitor cocktail (Roche Applied Science, Indianapolis, IN). The lysate was centrifuged for 10 min. at 20,000*g* at 4°C, and the supernatant transferred to a fresh tube. 200 µl of supernatant was mixed with 20 µl of protein G agarose beads (GE Healthcare Life Sciences, Piscataway, NJ) pre-bound with 5 µg of antibody in 800 µl mild lysis buffer. The agarose beads were then incubated for 1 h at 4°C with rocking, washed six times using mild lysis buffer and the bound proteins analyzed on immunoblots.

### *In vitro* Pulldown Assays

pMBP-Mage was previously described [36] and the control pMBP construct was supplied with a Maltose binding protein (MBP) purification kit (New England Biolabs, Ipswich, MA). Expression constructs were produced by inserting relevant full-length coding sequences into a Gateway pDEST-14 expression vector. MBP fused Mage (MBP-Mage) was expressed in *Escherichia coli* (ER2523, New England Biolabs) and immobilized onto amylose resin (E8200S) according to the manufacturer’s directions. ^35^S labeled probe proteins were expressed from Gateway pDest14 vectors using the TNT-coupled *in vitro* transcription-translation system (Promega, Madison, WI). For the *in vitro* binding assay, ^35^S-labeled probe proteins were incubated with immobilized MBP-Mage proteins in 500 µl of buffer (20 mM Tris, 100 mM NaCl, 0.5 mM EDTA, 10% glycerol, and 1% Tween-20, pH 7.6) containing 0.25% bovine serum albumin (BSA) and protease inhibitor cocktail [68] overnight at 4°C with end-over-end mixing. The resin was washed six times in 500 µl of the same buffer, and the bound proteins were resolved by SDS-PAGE and detected by autoradiography.

### Scanning Electron Microscopy (SEM) and Immunohistochemistry

Adult heads were prepared for SEM according to the HMDS method described in *Drosophila Protocols* [69] and imaged using a Scanning Electron Microscope (FEI (XL30), Philips, Hillsboro, OR). Dissection, fixation, BrdU labeling, and antibody staining of third larval instar eye-antennal discs were also carried out as described in *Drosophila Protocols.* Antibodies for immunohistochemistry included anti-cleaved caspase 3 (1∶1600 dilution, Cell Signaling Technologies, Beverly, MA), anti-BrdU (1∶200 dilution, Pharmingen San Jose, CA), and anti-phospho-histone H3 (Cell Signaling, 1∶1000 dilution). Secondary antibodies were used at a dilution of 1∶1000 (Alexa Fluor 488 and 586, Invitrogen). For the detection of apoptosis in third instar imaginal discs with an anti-cleaved caspase 3 antibody, embryos were collected at one hour intervals on grape juice plates and larvae were reared on yeast paste plates until the L3 molt. They were then transferred to 2 mM caffeine medium 32 h after the L3 molt and allowed to develop for a further 12 h before dissection. Images of the dissected discs were acquired using a LSM 700 confocal microscope (Carl Zeiss Inc., Thornwood, NY) and processed using Zen (Carl Zeiss). A maximum projection of all stacks of a confocal image was used to quantify the signal intensity of staining using a lower threshold to eliminate background staining. This value was divided by the area of each eye disc to obtain a ratio representing the relative amount of immunostaining. Data represent at least 7 eye discs per genotype per treatment.

## Supporting Information

Figure S1 **An ethyl methanesulfonate (EMS) screen for caffeine-sensitive mutants on chromosome 3R.** Ethylmethane sulfonate (EMS) mutagenized male flies carrying transgenic *FRT82B* sites were crossed en masse to *y,w; EGUF; FRT82B GMR-hid/TM3, Sb* virgin females in standard media. Non-*TM3, Sb* progeny males containing normal looking eyes were then collected and crossed in pools of 3–5 males to 3–5 *y,w; EGUF; FRT82B GMR-hid/TM3, Sb* virgin females in molasses and cornmeal media containing 2 mM caffeine. Non-*TM3, Sb* progeny males containing developmental defects in both eyes were selected and individually tested with *y,w; EGUF; FRT82B GMR-hid/TM3, Sb* virgin females in normal media to eliminate any false positive caffeine-independent mutations that might have arisen in the male germline. Once a caffeine-dependent phenotype was confirmed, the mutant was then crossed to *y,w; EGUF; FRT82B GMR-hid/TM3, Sb* virgin females to establish balanced stocks. “*” indicates a putative mutation.
(PDF)

Figure S2 **Caffeine sensitivity of** ***jnj*** **alleles is caused by loss of** ***Smc6*** **.** (A) mRNA transcript levels of *Smc6* and its neighboring genes *CHORD, CG5515* and *CG6204* in control and *jnj* mutant flies were measured by quantitative RT-PCR. All seven *jnj* alleles tested had reduced *Smc6* transcript levels ranging from 7% to 24% of the control level, while the transcript levels of the neighboring genes comparable to the control level. The caffeine screen starting stock “Iso” carrying the transgenic *FRT82B* site crossed to Df to normalize the *Smc6* level was used to generate control flies. “Df” is the deficiency chromosome *Df(3R)Exel6198*. (B) Knocking-down *Smc6* expression using RNAi in developing eye discs resulted in a caffeine-dependent adult rough eye phenotype. Control, *Eyeless-Gal4/+* was from a cross of *Eyeless-Gal4/Eyeless-Gal4* X *w^1118^* and *Smc6-*RNAi, *Eyeless-Gal4/+; UAS-Smc6-RNAi/+* resulted from the cross *Eyeless-Gal4/Eyeless-Gal4* X *UAS-Smc6-RNAi/+*. *UAS-Smc6-RNAi* was obtained from VDRC (#107055).
(PDF)

Figure S3 **Immunoblot for Mage.** Levels of endogenous Mage were measured in protein lysates from whole flies derived from various lines, immunoblotted with anti-Mage antibody. Genotypes were as follows: Lane 1: *sst^XL^/TM3,Sb*, 2: *sst^RZ^/TM3,Ser,ActGFP*, 3: *sst^XL^/sst^RZ^,* 4: *Df(3R)Antp1/TM3,Sb*, 5: *Df(3R)Antp1/sst^RZ^,* 6: *Df(3R)Antp1/sst^XL^*, 7. *w^1118^*, 8: *S2* cells, 9: *S2* cells dMAGE RNAi, 10: *sst^XL^*/TM3,Ser,ActGFP, 11: *sst^XL^/sst^XL^*, 12∶*3Kb+MAGE transgene/CyO; sst^XL^/sst^XL^.*
(PDF)

Figure S4 **Expression profiles of genes encoding** ***Smc5/6*** **complex proteins.** The expression profile figure for each gene was obtained from GEO Profiles database at NCBI (GDS2784) from the original data of Chintapalli *et al.* [40].
(PDF)

Figure S5 ***Smc6, MAGE*** **and** ***Smc5*** **mutants are sensitive to camptothecin, HU and MMS.** Flies eclosed from the same cross are indicated with a ‘□’. Embryos (n = 360, expected to be half homozygous or transheterozygous mutants and half heterozygous mutants) were collected from a given cross for each drug concentration and allowed to develop in media without or with each drug. Bars represent the survival index (*p*) ± SEM. Absence of a bar indicates that no flies survived at that drug concentration. The survival index was calculated by normalizing the number of eclosed adults from each drug treatment against the number of eclosed adults from the no treatment control. (A–C) *Smc6, MAGE* or *Smc5* homozygous, trans-heterozygous or hemizygous mutants have reduced survival when raised in media supplemented with 0.025 mM camptothecin; (D–F) *Smc6, MAGE* or *Smc5* homozygous, trans-heterozygous or hemizygous mutants have reduced survival when raised in media supplemented with hydroxyurea (HU); (G) *MAGE* mutants are sensitive to MMS; (H) *Smc5* mutants are sensitive to MMS. *Smc6* mutants are also sensitive to MMS (data not shown). *Smc6*: *R1* (*jnj^R1^*) and *X1* (*jnj^X1^*) are *Smc6* alleles. *Df* (*Df(3R)Exel6198*) is a deficiency chromosome uncovering the *Smc6* locus; *MAGE*: *RZ* (*sst^RZ^*) and *XL* (*sst^XL^*) are *MAGE* alleles. *Df* (*Df(3R)Antp^1^*) is a deficiency chromosome uncovering the *MAGE* locus. *Smc5*: *P5* (*Smc5^P{GSV1}GS3245^*) and *P7* (*Smc5^P{GSV6}GS14577^*) are *Smc5* alleles. *Df* (*Df(3L)BSC418*) is a deficiency chromosome uncovering the *Smc5* locus.
(PDF)

Figure S6 **Quantification the area of the adult eye as a measure of the genetic interaction of** ***MAGE*** **with** ***ATM*** **,** ***ATR*** **or** ***NBS1*** **.** *MAGE* (*EGUF/+; FRT82B sst^RZ^/FRT82B GMR-hid*, loss of *MAGE* in eye cells), *ey>ATM-RNAi* (knockdown of *ATM* in eye cells), *ey>ATR-RNAi* (knockdown of *ATR* in eye cells), *ey>NBS1-RNAi* (knockdown of *NBS1* in eye cells), *ey>ATM-RNAi;MAGE* (*EGUF/UAS-ATM-RNAi;FRT82B sst^RZ^*/*FRT82B GMR-hid,* loss of *MAGE* and knockdown of ATM in eye cells), *ey>ATR-RNAi;MAGE* (*EGUF/UAS-ATR-RNAi;FRT82B sst^RZ^*/*FRT82B GMR-hid,* loss of *MAGE* and knockdown of ATR in eye cells), and *ey>NBS1-RNAi;MAGE* (*EGUF/UAS-NBS1-RNAi;FRT82B sst^RZ^/FRT82B GMR-hid,* loss of *MAGE* and knockdown of *NBS1* in eye cells) flies were reared on either standard media or media containing 2 mM caffeine. A Student two-tailed *t*-test was performed to compare between genotypes.
(PDF)

Figure S7 ***NBS1*** **interacts with** ***MAGE*** **.** Representative eye phenotypes of *MAGE* (*EGUF/+; FRT82B sst^RZ^/FRT82B GMR-hid*, loss of *MAGE* in eye cells) and *ey>NBS1i* (knockdown of *NBS1* in eye cells) and *ey>NBS1i;MAGE* (*EGUF/UAS-NBS1-RNAi;FRT82B sst^RZ^/FRT82B GMR-hid,* loss of *MAGE* and knockdown of *NBS1* in eye cells) flies that were reared on either standard media or media containing 2 mM caffeine. The *EGUF* system carrying the *eyeless-Gal4* driver was used to drive the *UAS*-RNAi transgene in the eye and was also made the eyes homozygous for *sst^RZ^*.
(PDF)

Figure S8 ***Rad51 (SpnA-RNAi)*** **depletion rescues the** ***MAGE-RNAi*** **caffeine-sensitive eye phenotype.** Bars represent the percentage of flies with wildtype eye phenotypes among *MAGE* knockdown (*UAS-Drc2/+; UAS-MAGE-RNAi/+*) and *MAGE Rad51* double knockdown (*Drc2/+; UAS-MAGE-RNAi/UAS-SpnA-RNAi*) flies that were reared on either standard media or media containing 2 mM caffeine. Data were collected from 4 replicates of each cross. Absence of error bar indicates flies of this genotype had consistent phenotypes.
(PDF)

Table S1 ***sst*** **caffeine sensitivity can be rescued by a** ***MAGE*** **transgene.**
(PDF)

Table S2 **P-element excision of** ***P{GSV1}GS3245*** **and** ***P{GSV6}GS14577*** **produce both caffeine-sensitive and -insensitive lines.**
(PDF)

Table S3 **Caffeine sensitivity of** ***MAGE*** **and** ***Smc6*** **double mutants is similar to sensitivity of flies mutant for** ***Smc6*** **alone.**
(PDF)

Table S4 **Genes encoding Smc5/6 complexes in different model organisms.**
(PDF)

Table S5 ***mei-41/ATM*** **and** ***jnj/Smc6*** **double mutants have normal viability.**
(PDF)

Methods S1 **Supporting Methods.**
(PDF)
