# Supplementary figures and images for: The Smc5/Smc6/MAGE Complex Confers Resistance to Caffeine and Genotoxic Stress in Drosophila melanogaster
Source: PLoS One. 2013 Mar 28;8(3):e59866. doi: 10.1371/journal.pone.0059866 (PMC3610895; doi:10.1371/journal.pone.0059866)

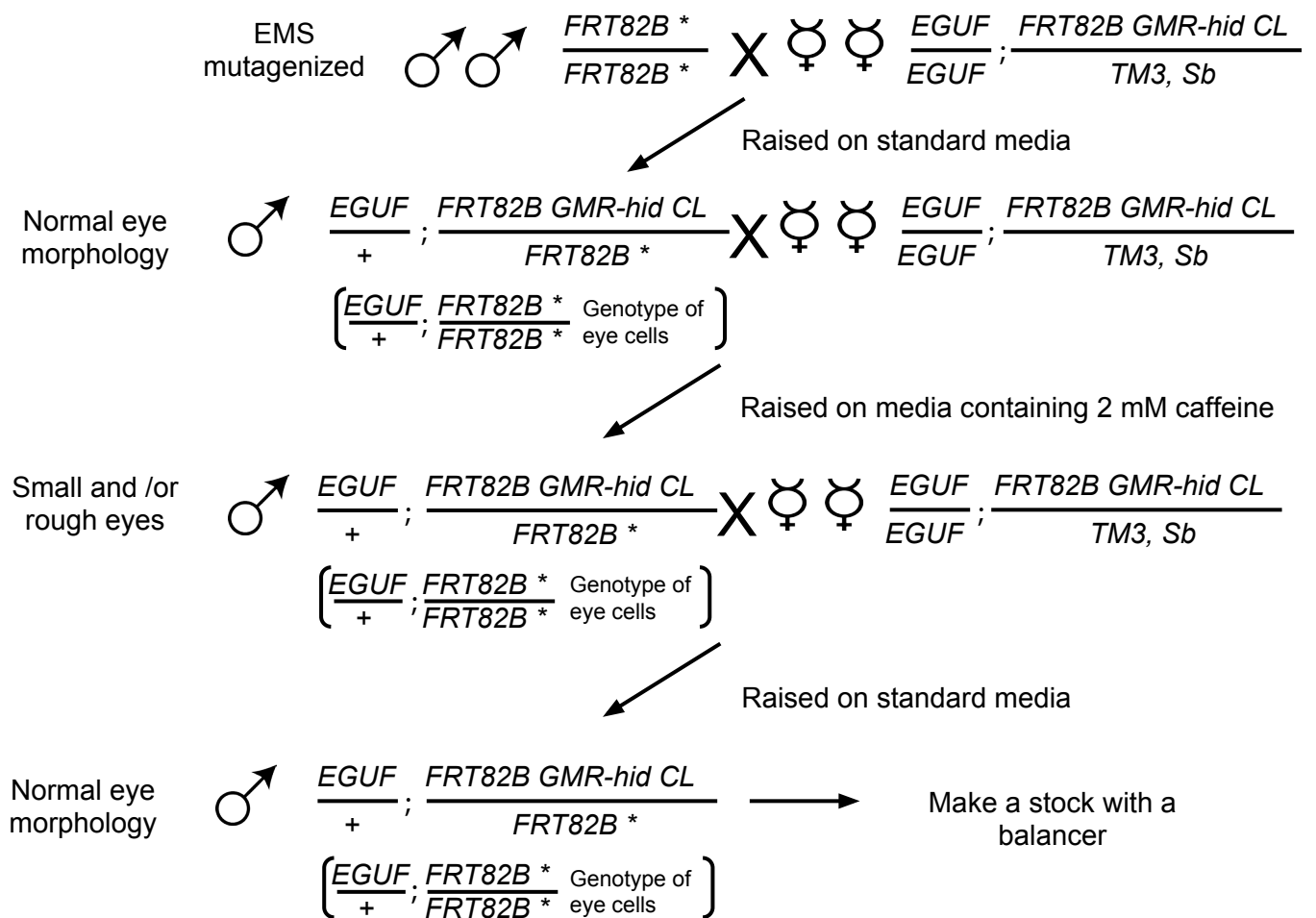

Fig. S1

Supplement: Figure S1 — An ethyl methanesulfonate (EMS) screen for caffeine-sensitive mutants on chromosome 3R. Ethylmethane sulfonate (EMS) mutagenized male flies carrying transgenic FRT82B sites were crossed en masse to y,w; EGUF; FRT82B GMR-hid/TM3, Sb virgin females in standard media. Non-TM3, Sb progeny males containing normal looking eyes were then collected and crossed in pools of 3–5 males to 3–5 y,w; EGUF; FRT82B GMR-hid/TM3, Sb virgin females in molasses and cornmeal media containing 2 mM caffeine. Non-TM3, Sb progeny males containing developmental defects in both eyes were selected and individually tested with y,w; EGUF; FRT82B GMR-hid/TM3, Sb virgin females in normal media to eliminate any false positive caffeine-independent mutations that might have arisen in the male germline. Once a caffeine-dependent phenotype was confirmed, the mutant was then crossed to y,w; EGUF; FRT82B GMR-hid/TM3, Sb virgin females to establish balanced stocks. “*” indicates a putative mutation. (PDF) [file pone.0059866.s001.pdf]

A

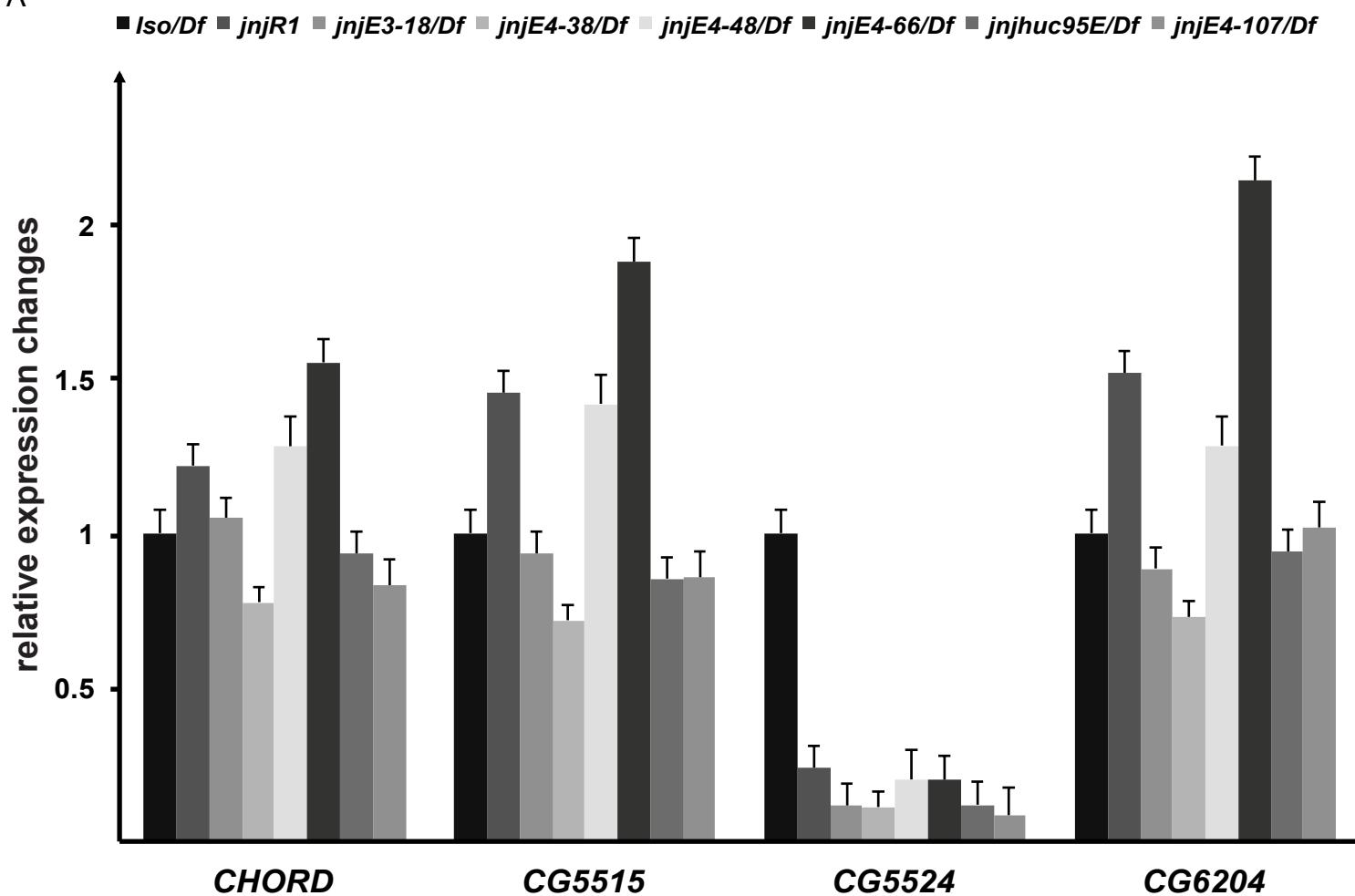

B

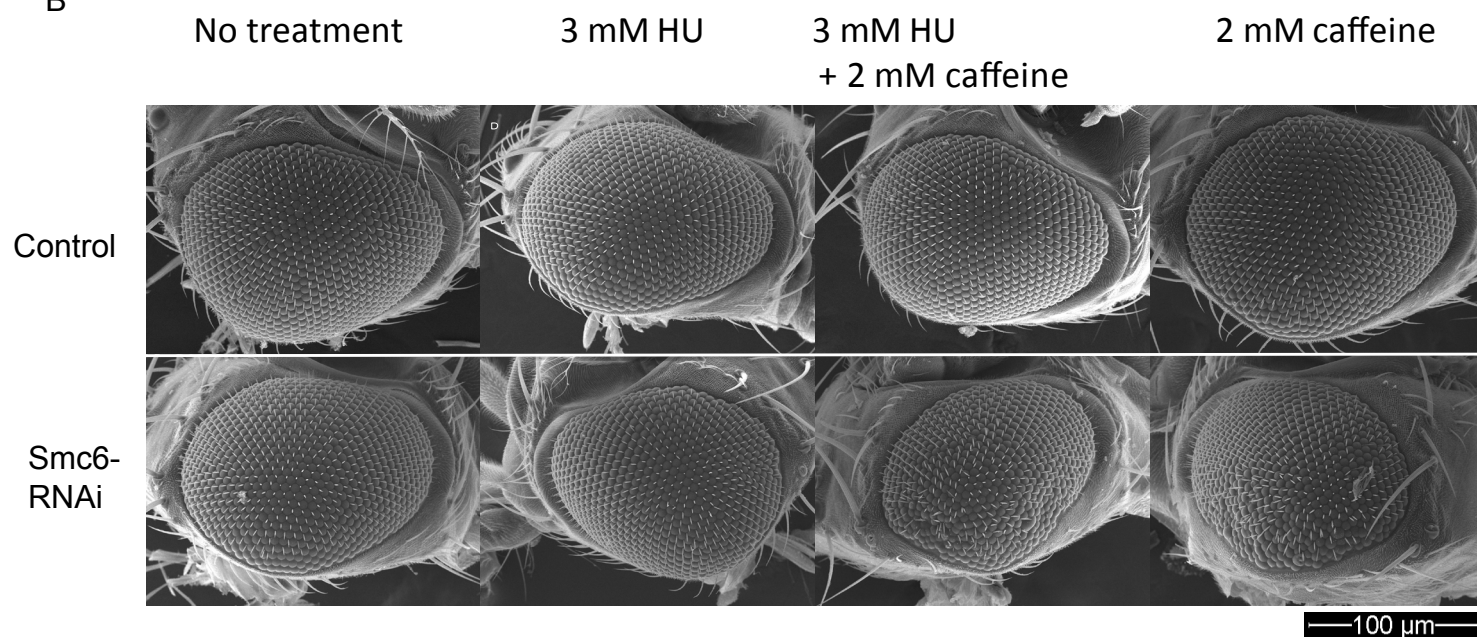

Fig. S2

Supplement: Figure S2 — Caffeine sensitivity of jnj alleles is caused by loss of Smc6 . (A) mRNA transcript levels of Smc6 and its neighboring genes CHORD, CG5515 and CG6204 in control and jnj mutant flies were measured by quantitative RT-PCR. All seven jnj alleles tested had reduced Smc6 transcript levels ranging from 7% to 24% of the control level, while the transcript levels of the neighboring genes comparable to the control level. The caffeine screen starting stock “Iso” carrying the transgenic FRT82B site crossed to Df to normalize the Smc6 level was used to generate control flies. “Df” is the deficiency chromosome Df(3R)Exel6198. (B) Knocking-down Smc6 expression using RNAi in developing eye discs resulted in a caffeine-dependent adult rough eye phenotype. Control, Eyeless-Gal4/+ was from a cross of Eyeless-Gal4/Eyeless-Gal4 X w1118 and Smc6-RNAi, Eyeless-Gal4/+; UAS-Smc6-RNAi/+ resulted from the cross Eyeless-Gal4/Eyeless-Gal4 X UAS-Smc6-RNAi/+. UAS-Smc6-RNAi was obtained from VDRC (#107055). (PDF) [file pone.0059866.s002.pdf]

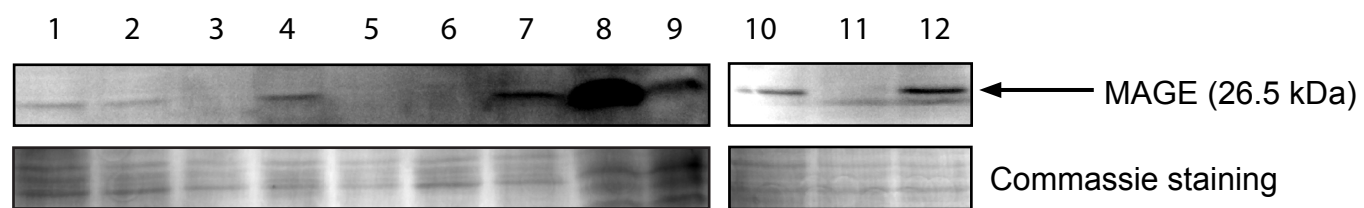

Fig. S3

Supplement: Figure S3 — Immunoblot for Mage. Levels of endogenous Mage were measured in protein lysates from whole flies derived from various lines, immunoblotted with anti-Mage antibody. Genotypes were as follows: Lane 1: sstXL/TM3,Sb, 2: sstRZ/TM3,Ser,ActGFP, 3: sstXL/sstRZ, 4: Df(3R)Antp1/TM3,Sb, 5: Df(3R)Antp1/sstRZ, 6: Df(3R)Antp1/sstXL, 7. w1118, 8: S2 cells, 9: S2 cells dMAGE RNAi, 10: sstXL/TM3,Ser,ActGFP, 11: sstXL/sstXL, 12∶3Kb+MAGE transgene/CyO; sstXL/sstXL. (PDF) [file pone.0059866.s003.pdf]

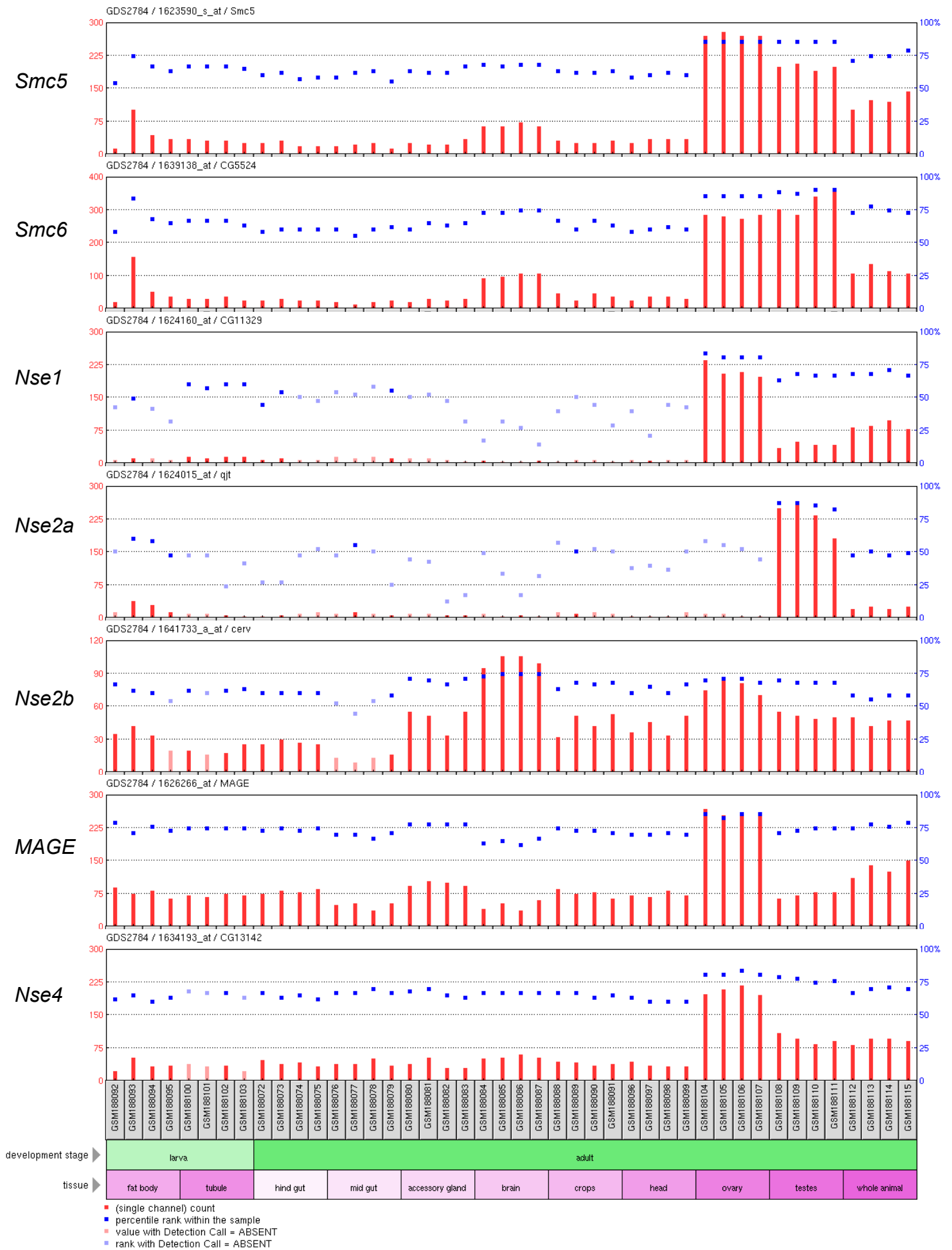

Fig. S4

Supplement: Figure S4 — Expression profiles of genes encoding Smc5/6 complex proteins. The expression profile figure for each gene was obtained from GEO Profiles database at NCBI (GDS2784) from the original data of Chintapalli et al. [40]. (PDF) [file pone.0059866.s004.pdf]

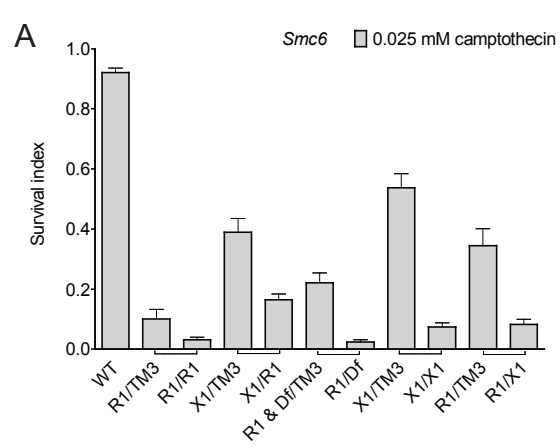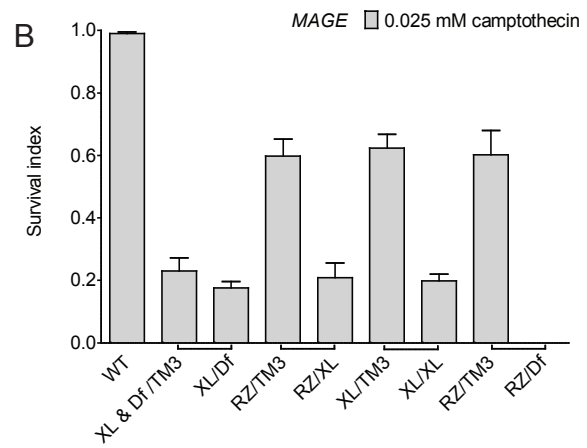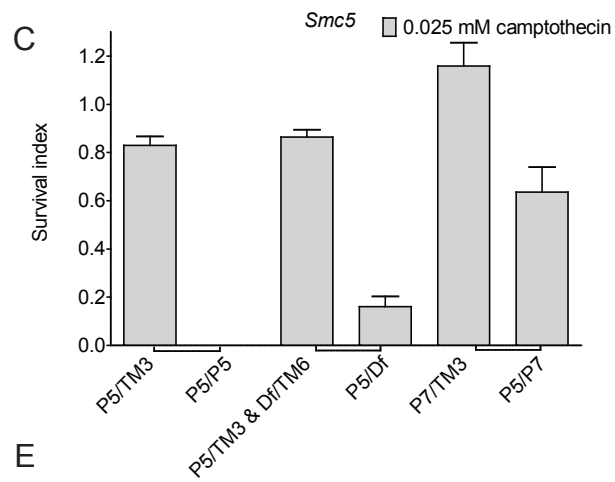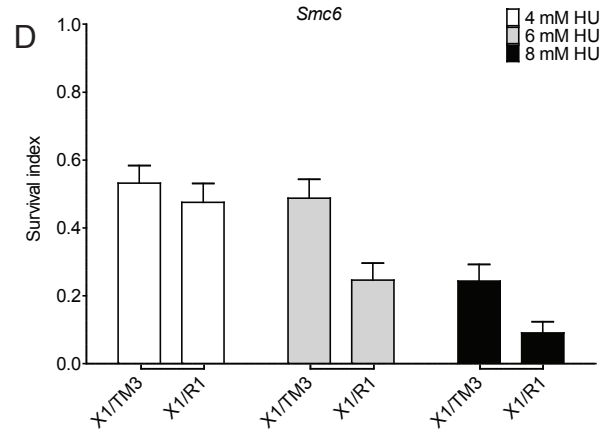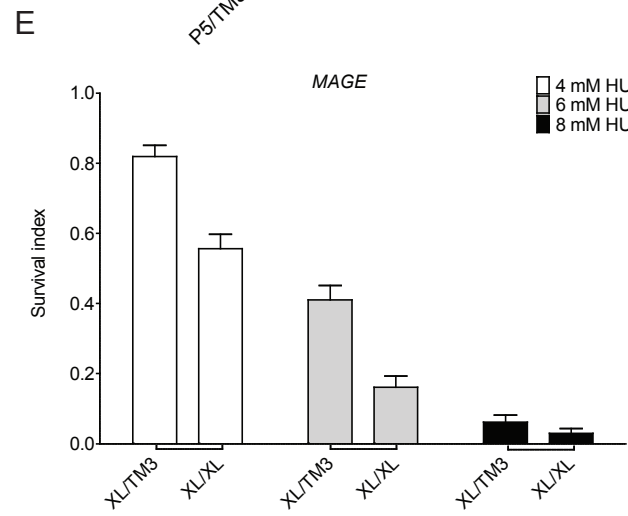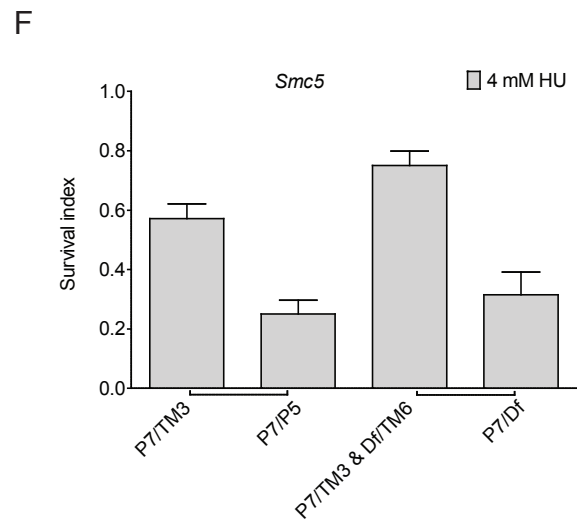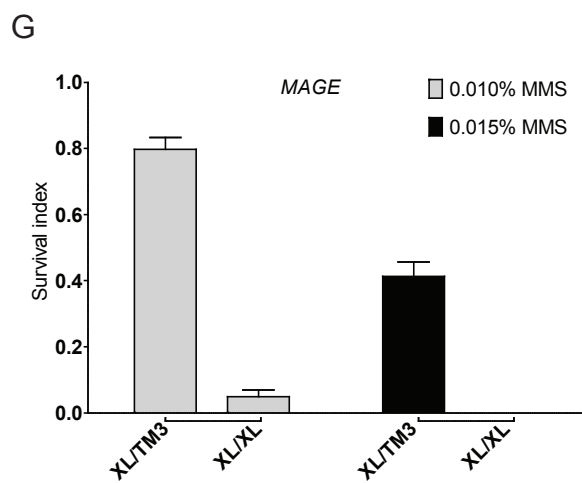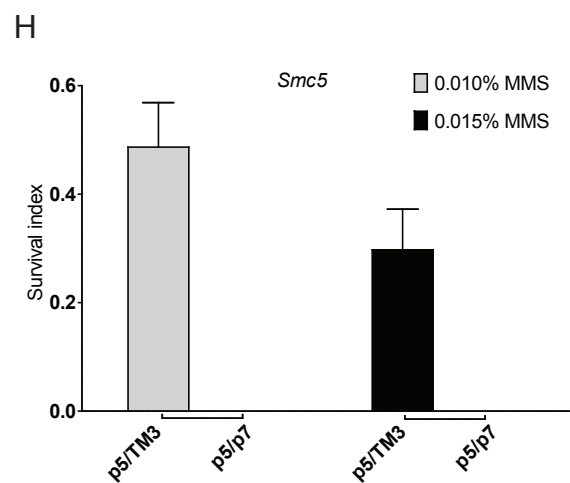

Supplement: Figure S5 — Smc6, MAGE and Smc5 mutants are sensitive to camptothecin, HU and MMS. Flies eclosed from the same cross are indicated with a ‘□’. Embryos (n = 360, expected to be half homozygous or transheterozygous mutants and half heterozygous mutants) were collected from a given cross for each drug concentration and allowed to develop in media without or with each drug. Bars represent the survival index (p) ± SEM. Absence of a bar indicates that no flies survived at that drug concentration. The survival index was calculated by normalizing the number of eclosed adults from each drug treatment against the number of eclosed adults from the no treatment control. (A–C) Smc6, MAGE or Smc5 homozygous, trans-heterozygous or hemizygous mutants have reduced survival when raised in media supplemented with 0.025 mM camptothecin; (D–F) Smc6, MAGE or Smc5 homozygous, trans-heterozygous or hemizygous mutants have reduced survival when raised in media supplemented with hydroxyurea (HU); (G) MAGE mutants are sensitive to MMS; (H) Smc5 mutants are sensitive to MMS. Smc6 mutants are also sensitive to MMS (data not shown). Smc6: R1 (jnjR1) and X1 (jnjX1) are Smc6 alleles. Df (Df(3R)Exel6198) is a deficiency chromosome uncovering the Smc6 locus; MAGE: RZ (sstRZ) and XL (sstXL) are MAGE alleles. Df (Df(3R)Antp1) is a deficiency chromosome uncovering the MAGE locus. Smc5: P5 (Smc5P{GSV1}GS3245) and P7 (Smc5P{GSV6}GS14577) are Smc5 alleles. Df (Df(3L)BSC418) is a deficiency chromosome uncovering the Smc5 locus. (PDF) [file pone.0059866.s005.pdf]

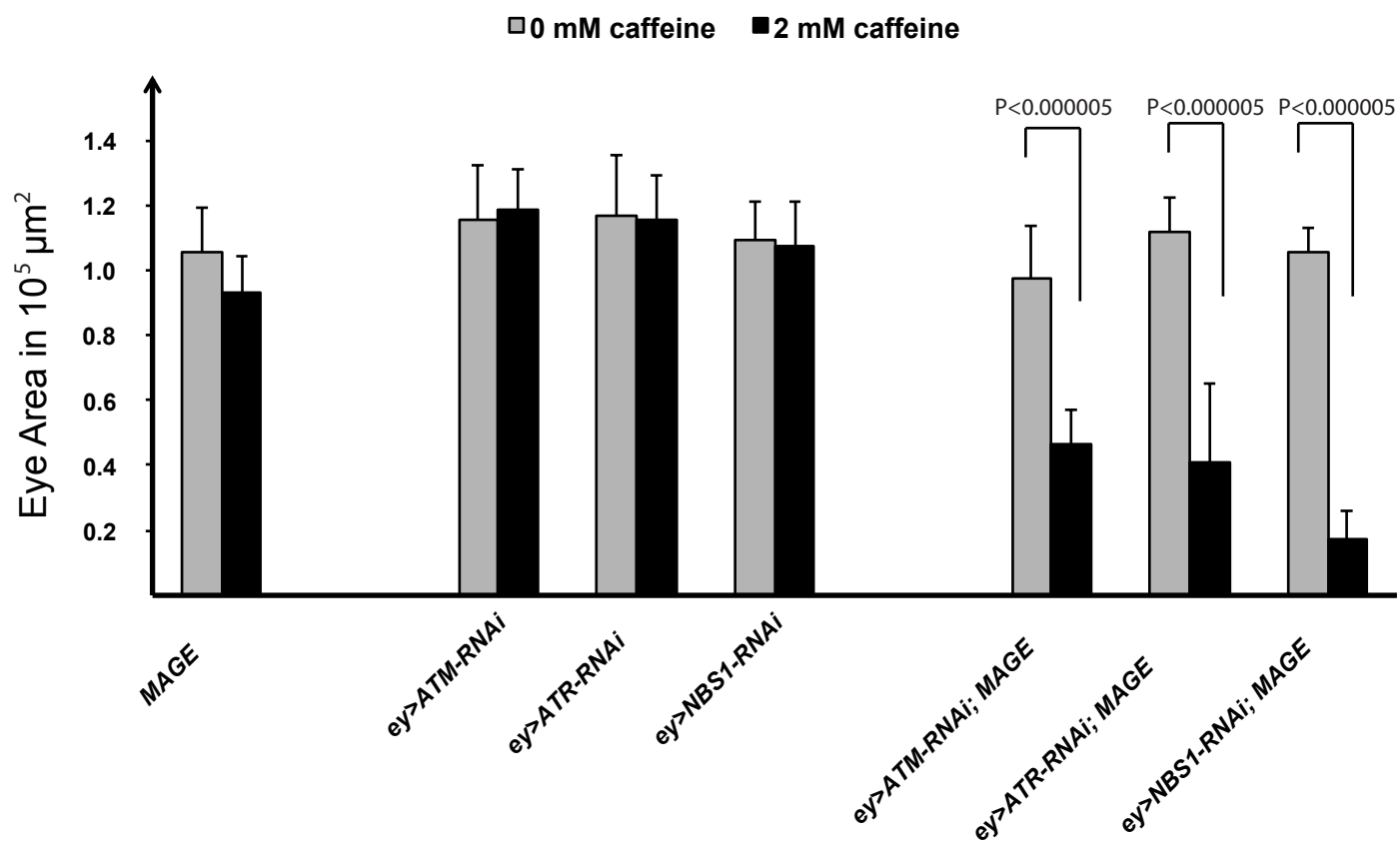

Fig. S6

Supplement: Figure S6 — Quantification the area of the adult eye as a measure of the genetic interaction of MAGE with ATM , ATR or NBS1 . MAGE (EGUF/+; FRT82B sstRZ/FRT82B GMR-hid, loss of MAGE in eye cells), ey>ATM-RNAi (knockdown of ATM in eye cells), ey>ATR-RNAi (knockdown of ATR in eye cells), ey>NBS1-RNAi (knockdown of NBS1 in eye cells), ey>ATM-RNAi;MAGE (EGUF/UAS-ATM-RNAi;FRT82B sstRZ/FRT82B GMR-hid, loss of MAGE and knockdown of ATM in eye cells), ey>ATR-RNAi;MAGE (EGUF/UAS-ATR-RNAi;FRT82B sstRZ/FRT82B GMR-hid, loss of MAGE and knockdown of ATR in eye cells), and ey>NBS1-RNAi;MAGE (EGUF/UAS-NBS1-RNAi;FRT82B sstRZ/FRT82B GMR-hid, loss of MAGE and knockdown of NBS1 in eye cells) flies were reared on either standard media or media containing 2 mM caffeine. A Student two-tailed t-test was performed to compare between genotypes. (PDF) [file pone.0059866.s006.pdf]

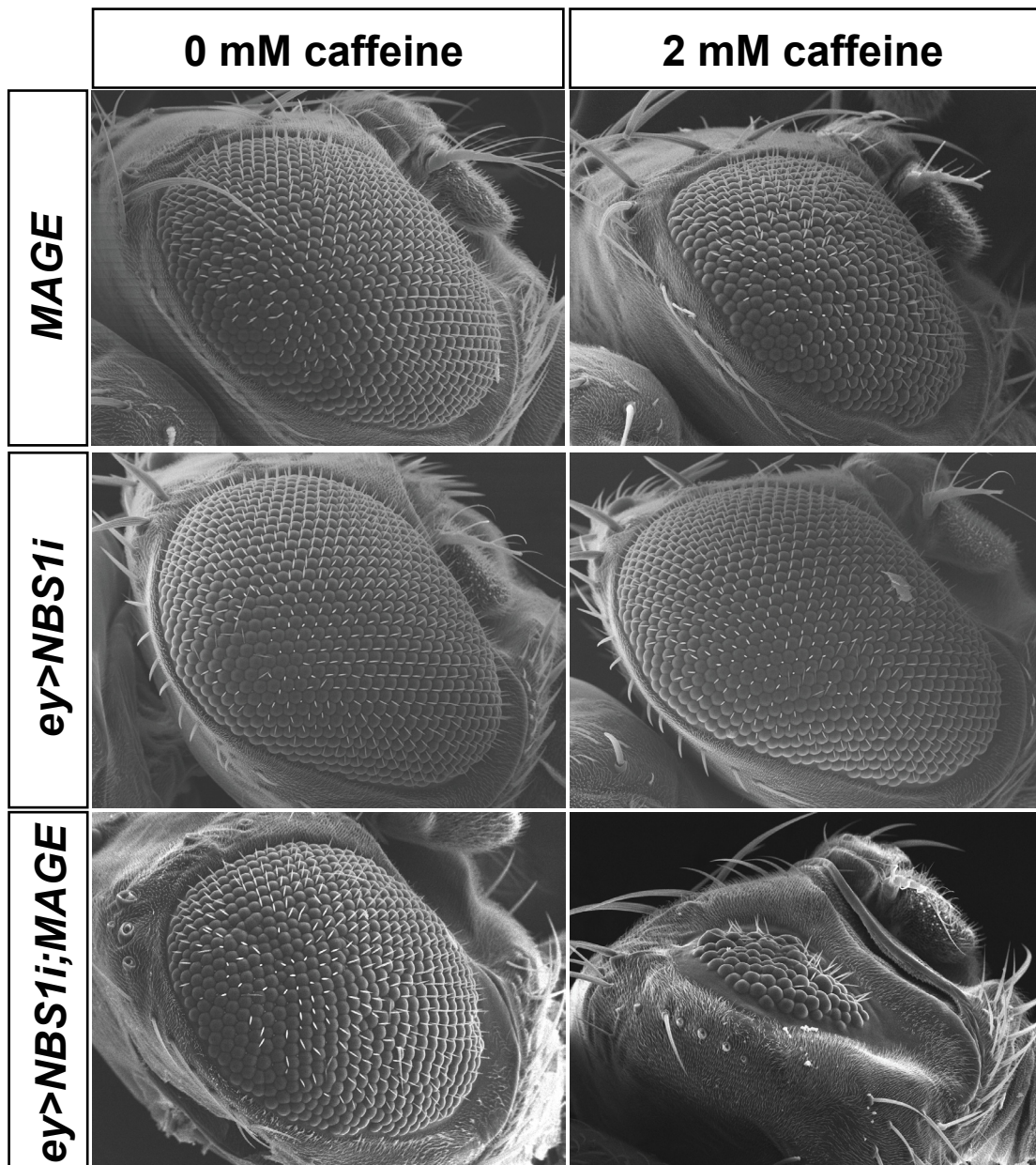

Fig. S7

Supplement: Figure S7 — NBS1 interacts with MAGE . Representative eye phenotypes of MAGE (EGUF/+; FRT82B sstRZ/FRT82B GMR-hid, loss of MAGE in eye cells) and ey>NBS1i (knockdown of NBS1 in eye cells) and ey>NBS1i;MAGE (EGUF/UAS-NBS1-RNAi;FRT82B sstRZ/FRT82B GMR-hid, loss of MAGE and knockdown of NBS1 in eye cells) flies that were reared on either standard media or media containing 2 mM caffeine. The EGUF system carrying the eyeless-Gal4 driver was used to drive the UAS-RNAi transgene in the eye and was also made the eyes homozygous for sstRZ. (PDF) [file pone.0059866.s007.pdf]

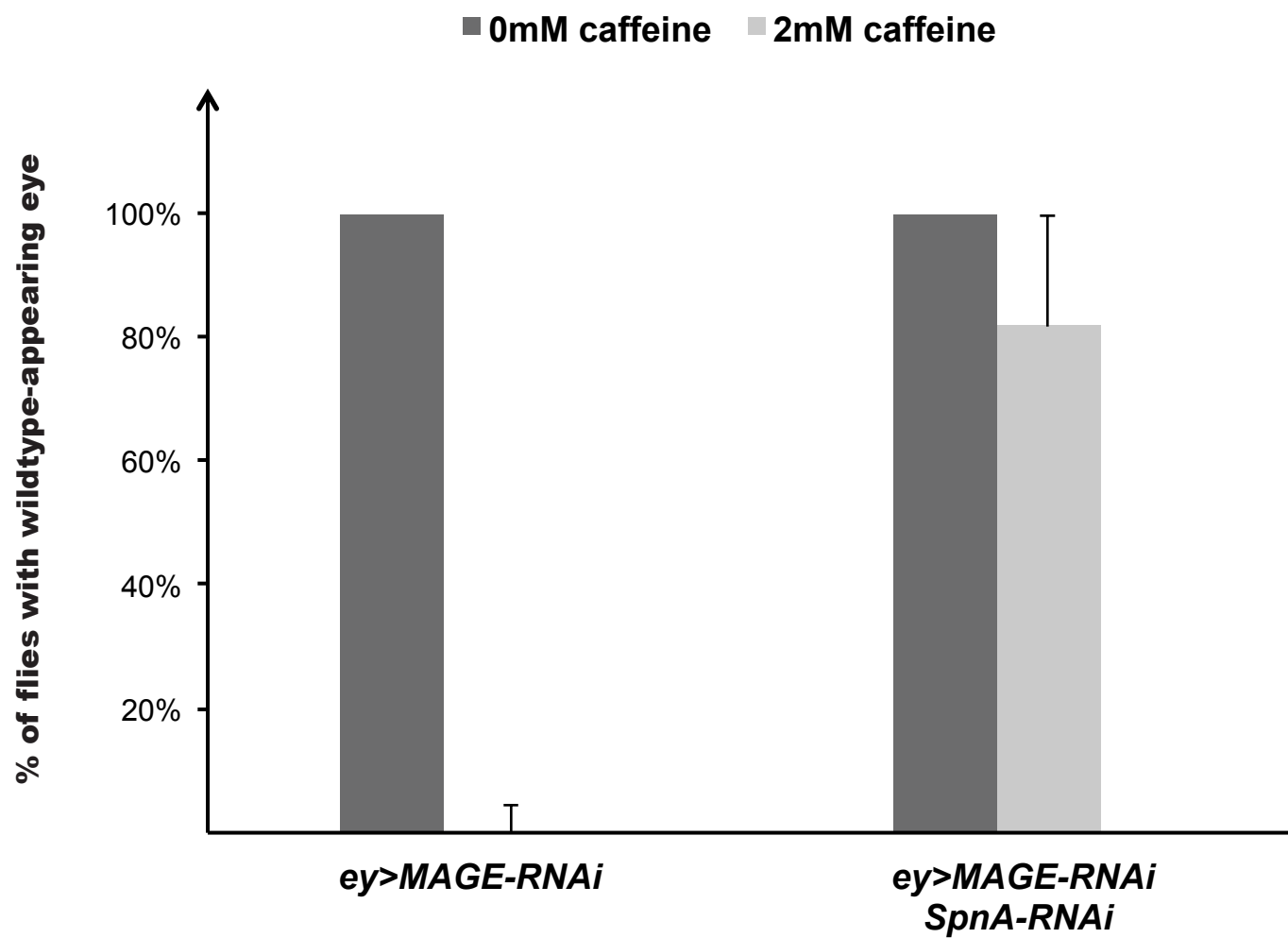

Fig. S8

Supplement: Figure S8 — Rad51 (SpnA-RNAi) depletion rescues the MAGE-RNAi caffeine-sensitive eye phenotype. Bars represent the percentage of flies with wildtype eye phenotypes among MAGE knockdown (UAS-Drc2/+; UAS-MAGE-RNAi/+) and MAGE Rad51 double knockdown (Drc2/+; UAS-MAGE-RNAi/UAS-SpnA-RNAi) flies that were reared on either standard media or media containing 2 mM caffeine. Data were collected from 4 replicates of each cross. Absence of error bar indicates flies of this genotype had consistent phenotypes. (PDF) [file pone.0059866.s008.pdf]
