# Supplementary material for: The Smc5/Smc6/MAGE Complex Confers Resistance to Caffeine and Genotoxic Stress in Drosophila melanogaster
Source: PLoS One. 2013 Mar 28;8(3):e59866. doi: 10.1371/journal.pone.0059866 (PMC3610895; doi:10.1371/journal.pone.0059866)
Supplement: Table S1 — sst caffeine sensitivity can be rescued by a MAGE transgene. (PDF) [file pone.0059866.s009.pdf]

Table S1. *sst* caffeine sensitivity can be rescued by a *MAGE* transgene.

Genotype	0 mM caffeine	2 mM caffeine
3Kb+MAGE/+;sst^{XL}/sst^{XL}	64	118
3Kb+MAGE/+; sst^{XL}/TM3, Ser, ActGFP	59	77
CyO/+; sst^{XL}/sst^{XL}	52	0
CyO/+; sst^{XL}/TM3, Ser, ActGFP	77	30

All genotypes were produced from cross *sst^{XL}/sst^{XL}* X *3Kb+dMAGE/CyO;sst^{XL}/TM3, Ser, ActGFP*.
