# Supplementary material for: The Smc5/Smc6/MAGE Complex Confers Resistance to Caffeine and Genotoxic Stress in Drosophila melanogaster
Source: PLoS One. 2013 Mar 28;8(3):e59866. doi: 10.1371/journal.pone.0059866 (PMC3610895; doi:10.1371/journal.pone.0059866)
Supplement: Table S2 — P-element excision of P{GSV1}GS3245 and P{GSV6}GS14577 produce both caffeine-sensitive and -insensitive lines. (PDF) [file pone.0059866.s010.pdf]

Table S2. P-element excision of *P{GSV1}GS3245* and *P{GSV6}GS14577* produce both caffeine-sensitive and -insensitive lines.

Suppl. Table 2 A

Excision alleles	Caffeine sensitive?	$Smc5^{P14577}$/$Smc5^{P14577}$ X $Smc5^{exP14577}$/TM3,Sb				$Smc5^{exP14577}$/$Smc5^{exP14577}$ X $Smc5^{Df(3L)BSC418}$/TM6,Sb			
		Standard media		Caffeine 1mM		Standard media		Caffeine 1mM	
		$Smc5^{exP14577}$/$Smc5^{P14577}$	$Smc5^{P14577}$/TM3,Sb	$Smc5^{exP14577}$/$Smc5^{P14577}$	$Smc5^{P14577}$/TM3,Sb	$Smc5^{exP14577}$/$Smc5^{Df(3L)BSC418}$	$Smc5^{exP14577}$/TM6,Sb	$Smc5^{exP14577}$/$Smc5^{Df(3L)BSC418}$	$Smc5^{exP14577}$/TM6,Sb
1	No	50	56	18	27	61	97	51	57
2	No	28	33	14	18	60	81	30	47
3	Yes	58	79	0	16	51	30	0	11
4	No	62	101	19	35	30	33	29	36
5	Yes	N/A	N/A	N/A	N/A	42	56	0	12
7	Yes	52	77	0	22	39	40	0	8
9	Yes	24	19	N/A	N/A	41	56	1	24
10	Yes	29	40	0	2	45	47	0	16
11	Yes	57	53	0	43	38	47	1	24
12	No	17	30	15	13	63	77	26	39
13	No	54	90	28	35	33	43	14	35
14	Yes	54	61	0	21	29	32	0	33
15	Yes	52	74	0	20	82	84	11	27
Original Allele		$Smc5^{P14577}$/$Smc5^{P14577}$ X $Smc5^{P14577}$/TM3,TM3,Ser,ActGFP				$Smc5^{P14577}$/$Smc5^{P14577}$ X $Smc5^{Df(3L)BSC418}$/TM6,Sb			
		$Smc5^{P14577}$/$Smc5^{P14577}$	$Smc5^{P14577}$/TM3,Ser,ActGFP	$Smc5^{P14577}$/$Smc5^{P14577}$	$Smc5^{P14577}$/TM3,Ser,ActGFP	$Smc5^{P14577}$/$Smc5^{Df(3L)BSC418}$	$Smc5^{P14577}$/TM6,Sb	$Smc5^{P14577}$/$Smc5^{Df(3L)BSC418}$	$Smc5^{P14577}$/TM6,Sb
	Yes	58	77	0	33	44	56	0	7

Suppl. Table 2 B

Excision alleles	Caffeine sensive?	$Smc5^{P3245}$/$Smc5^{P3245}$ X $Smc5^{exP3245}$/TM3,Sb				$Smc5^{exP3245}$/TM3,Sb X $Smc5^{Df(3L)BSC418}$/TM6,Sb			
		Standard media		Caffeine 1mM		Standard media		Caffeine 1mM	
		$Smc5^{exP3245}$/$Smc5^{P3245}$	$Smc5^{P3245}$/TM3,Sb	$Smc5^{exP3245}$/$Smc5^{P3245}$	$Smc5^{P3245}$/TM3,Sb	$Smc5^{exP3245}$/$Smc5^{Df(3L)BSC418}$	$Smc5^{exP3245\ or\ Df(3L)BSC418}$/TM6 or TM3,Sb	$Smc5^{exP3245}$/$Smc5^{Df(3L)BSC418}$	$Smc5^{exP3245\ or\ Df(3L)BSC418}$/TM6 or TM3,Sb
1	No	10	32	13	34	46	82	27	45
2	No	15	72	17	41	57	119	22	45

3	No	19	53	6	46	N/A	N/A	N/A	N/A
4	No	39	115	6	39	29	64	12	44
5	No	37	128	17	49	30	117	15	48
6	No	50	108	25	40	56	109	18	28
7	No	20	72	31	52	43	64	24	28
8	No	34	106	0	69	37	95	0	34
9	Yes	27	155	0	73	55	116	0	36
10	Yes	44	105	26	30	57	87	16	50
11	No	42	127	22	56	35	147	10	39
12	Yes	20	77	0	51	30	110	0	30
13	Yes	34	84	0	48	11	33	0	33
Original Alleles		*Smc5*^{P3245}/Smc5^{P3245} X Smc5^{P3245}/TM3,TM3,Ser,ActGFP				*Smc5*^{P3245}/TM3,Sb X Smc5$^{Df(3L)BSC418}$/TM6,Sb			
		Smc5^{P3245}/Smc5^{P3245}	*Smc5^{P3245}/TM3,Ser,ActGFP*	*Smc5^{P3245}/Smc5^{P3245}*	*Smc5^{P3245}/TM3,Ser,ActGFP*	*Smc5^{P3245}/Smc5$^{Df(3L)BSC418}$*	*Smc5$^{P3245\ or\ Df(3L)BSC418}$/TM6 or TM3,Sb*	*Smc5^{P3245}/Smc5$^{Df(3L)BSC418}$*	*Smc5$^{P3245\ or\ Df(3L)BSC418}$/TM6 or TM3,Sb*
	Yes	30	71	0	52	39	96	0	46
