# Supplementary material for: The Smc5/Smc6/MAGE Complex Confers Resistance to Caffeine and Genotoxic Stress in Drosophila melanogaster
Source: PLoS One. 2013 Mar 28;8(3):e59866. doi: 10.1371/journal.pone.0059866 (PMC3610895; doi:10.1371/journal.pone.0059866)
Supplement: Table S3 — Caffeine sensitivity of MAGE and Smc6 double mutants is similar to sensitivity of flies mutant for Smc6 alone. (PDF) [file pone.0059866.s011.pdf]

Table S3. Caffeine sensitivity of *MAGE* and *Smc6* double mutants is similar to sensitivity of flies mutant for *Smc6* alone.

Cross	Genotype	Mutant status	No caffeine	Caffeine	Sensitivity ratio
1	$sst^{XL}, jnj^{X1}/sst^{XL}, jnj^{R1}$	Double Mutant	104	9	0.07
	$sst^{XL}, jnj^{R1\ or\ X1}$/*TM3,Ser,ActGFP*	Double Het	226	267	
2	$+, jnj^{X1}/sst^{XL}, jnj^{R1}$	Smc6 Mutant	224	8	0.05
	$+, jnj^{X1}$/*TM3,Ser,ActGFP*	Smc6 Het	238	189	
3	$sst^{XL}, +/sst^{XL}, jnj^{R1}$	MAGE Mutant	279	83	0.34
	sst^{XL}, +/*TM3,Ser,ActGFP*	MAGE Het	310	274	

Caffeine sensitivity of *MAGE* and *Smc6* double mutants or single mutants was tested using media with 0.25 mM caffeine. A Sensitivity ratio was calculated by dividing the ratio of the homozygous versus the heterozygous flies surviving on media containing caffeine by the ratio of homozygous to heterozygous flies surviving on standard media. A Sensitivity ratio of 1 indicates that caffeine has no effect. Double Mutant: both *sst*/*MAGE* and *jnj/Smc6* inactivated; Double Het: *MAGE* and *Smc6* heterozygous mutant; Smc6 Mutant: *Smc6* inactivated; MAGE Mutant: *MAGE* inactivated; Het: heterozygous for either *MAGE* or *Smc6*.
