# Supplementary material for: The Smc5/Smc6/MAGE Complex Confers Resistance to Caffeine and Genotoxic Stress in Drosophila melanogaster
Source: PLoS One. 2013 Mar 28;8(3):e59866. doi: 10.1371/journal.pone.0059866 (PMC3610895; doi:10.1371/journal.pone.0059866)
Supplement: Table S4 — Genes encoding Smc5/6 complexes in different model organisms. (PDF) [file pone.0059866.s012.pdf]

Table S4. Genes encoding Smc5/6 complexes in different model organisms.

component	*Drosophila* CG	*Drosophila* symbol in FlyBase	*Schizosaccharomyces pombe* gene	*Saccharomyces cerevisiae* gene	Human gene
Smc5	CG32438	Dmel*Smc5*	Spr18	YOL034W/SMC5	SMC5L1<br>(alias KIAA0594, SMC5L1)
Smc6	CG5524	Dmel\CG5524	Rad18	YLR383W/SMC6<br>Rhc18	SMC6L1<br>(alias FLJ22116, FLJ35534, SMC-6, SMC6L1, hSMC6)
Nse1	CG11329	Dmel\CG11329	NSE1	YLR007W/NSE1	NSMCE1<br>(alias HSPC333, NSE1)
Nse2	CG13732<br>CG15645	*Dmel\qjt<br>Dmel\cerv	NSE2	YEL019C/MMS21	NSMCE2<br>(alias C8orf36, FLJ32440, MMS21, NSE2)
Nse3	CG10059	Dmel*MAGE*<br>(alias dMAGE)		YDR288W/NSE3	All MAGE genes<br>NDNL2<br>(alias HCA4, MAGEG1, MAGEL3, NSE3, NSMCE3)
Nse4	CG13142	Dmel\CG13142	Rad 62	YDL105W/NSE4<br>(alias Qri2)	NSMCE4A<br>(alias NSE4APP4762, C10orf86, FLJ20003, NSE4A)<br><br>NSMCE4B<br>(alias NSE4B, EID3)/<br>EID1

* used by Chiolo I, Minoda A, Colmenares SU, Polyzos A, Costes SV, Karpen GH. 2011. Double-strand breaks in heterochromatin move outside of a dynamic HP1a domain to complete recombinational repair. Cell 144:732-744.

Nse= Non-SMC (structural maintenance of chromosomes) element 1 protein; SMC= structural maintenance of chromosomes
