# Supplementary material for: The Smc5/Smc6/MAGE Complex Confers Resistance to Caffeine and Genotoxic Stress in Drosophila melanogaster
Source: PLoS One. 2013 Mar 28;8(3):e59866. doi: 10.1371/journal.pone.0059866 (PMC3610895; doi:10.1371/journal.pone.0059866)
Supplement: Table S5 — mei-41/ATM and jnj/Smc6 double mutants have normal viability. (PDF) [file pone.0059866.s013.pdf]

Table S5. ***mei-41/ATM*** **and** ***jnj/Smc6*** **double mutants have normal viability.**

	$jnj^{X1}/jnj^{Df(3R)Exel6198}$	jnj^{X1}/*TM3,Ser,ActGFP*
$mei\text{-}41^{D3}/Y$	99^{ns}	79^{ns}
FM7/Y	22^{***}	35^{***}
$mei\text{-}41^{D3}/+$	118^{ns}	110 (double heterozygotes)
FM7/+	118^{ns}	105^{ns}

jnj^{X1} homozygous males were crossed to $mei\text{-}41^{D3}$/*FM7*; $jnj^{Df(3R)Exel6198}$/*TM3,Ser,ActGFP*. The progeny representing the eight possible genotypes were counted. The number of progeny for each genotype was compared with the number of progeny heterozygotes for *mei41* and *Smc6* ($mei41^{D3}/+$; jnj^{X1}/*TM3,Ser,ActGFP*) using a chi-square test, with equal numbers expected in each category. "ns" indicates the number of progeny was not significantly different from the number of double heterozygotes ($P > 0.05$) while "***" indicates the number of progeny was significantly different from the number of double heterozygotes ($P < 0.001$). Fewer *FM7/Y* progeny survived, independent of *jnj* genotype, presumably because of non-balanced mutations on the *FM7* chromosome that reduce viability.
