# Supplementary material for: The Smc5/Smc6/MAGE Complex Confers Resistance to Caffeine and Genotoxic Stress in Drosophila melanogaster
Source: PLoS One. 2013 Mar 28;8(3):e59866. doi: 10.1371/journal.pone.0059866 (PMC3610895; doi:10.1371/journal.pone.0059866)
Supplement: Methods S1 — Supporting Methods. (PDF) [file pone.0059866.s014.pdf]

Supporting Methods
Li et al., PLoS Genetics

**Quantitative RT-PCR.** Total RNA was extracted from adult flies using Trizol reagent (Invitrogen). RNA concentration and integrity were determined by a Nanodrop ND-1000 (NanoDrop products, Wilmington, DE) and Agilent 2100 Bioanalyser (Agilent Technologies, Santa Clara, CA), respectively. One µg of total RNA per reaction was used for double strand cDNA synthesis (Applied Biosystems). Then, 2.5 µl of 1/20 diluted cDNA was used for each qPCR reaction with quantification based on SYBR Green incorporation (Applied Biosystems).
